# Supplementary material for: Second-Generation JK-206 Targets the Oncogenic Signal Mediator RHOA in Gastric Cancer
Source: Cancers (Basel). 2022 Mar 22;14(7):1604. doi: 10.3390/cancers14071604 (PMC8997135; doi:10.3390/cancers14071604)
Supplement: Supplementary file 1 [file cancers-14-01604-s001.zip › cancers-1637280-supplementary.pdf]

Second-generation JK-206 targets the oncogenic signal mediator RHOA in gastric cancer

Myeonghun Beak, Sungjin Park, and Jin-Hee Kim et al.

Supplementary data

Contents

Figure S1. RHOA inhibitors suppress cell growth and migration in GC.

Figure S2. Cell viability assay at 2 μM treatment.

Figure S3. Expression patterns of the DEGs (depicted in Figure 4b) in JK-206, JK-312, and DMSO treatments in GC cells.

Figure S4. JK-206 and JK-312 treatments in GC cells down-regulated hallmark gene sets of Myc targets, G2/M checkpoint, and E2F targets compared to the DMSO treatment in GC cells.

Figure S5. Expression patterns of the DEGs (depicted in Figure 4b) by another RHOA inhibitors in GC cells.

Figure S6. Correlations with *RHOA* and DEGs (depicted in Figure 4b) and expression patterns of the DEGs by knockdown and knockout of *RHOA* in GC and prostate cancer cells.

Figure S7. Differentially expressed genes in association with *RHOA* expression level in an independent GC dataset (GSE36968).

Method S1. Synthesis of hydrazide derivatives

Table S1. A list of DEGs common and uncommon to JK-206 treated (versus DMSO treated) and JK-312 treated (versus DMSO treated) GC cells.

Cell viability assay
(Rhosin, JK-208 ~ JK-214)

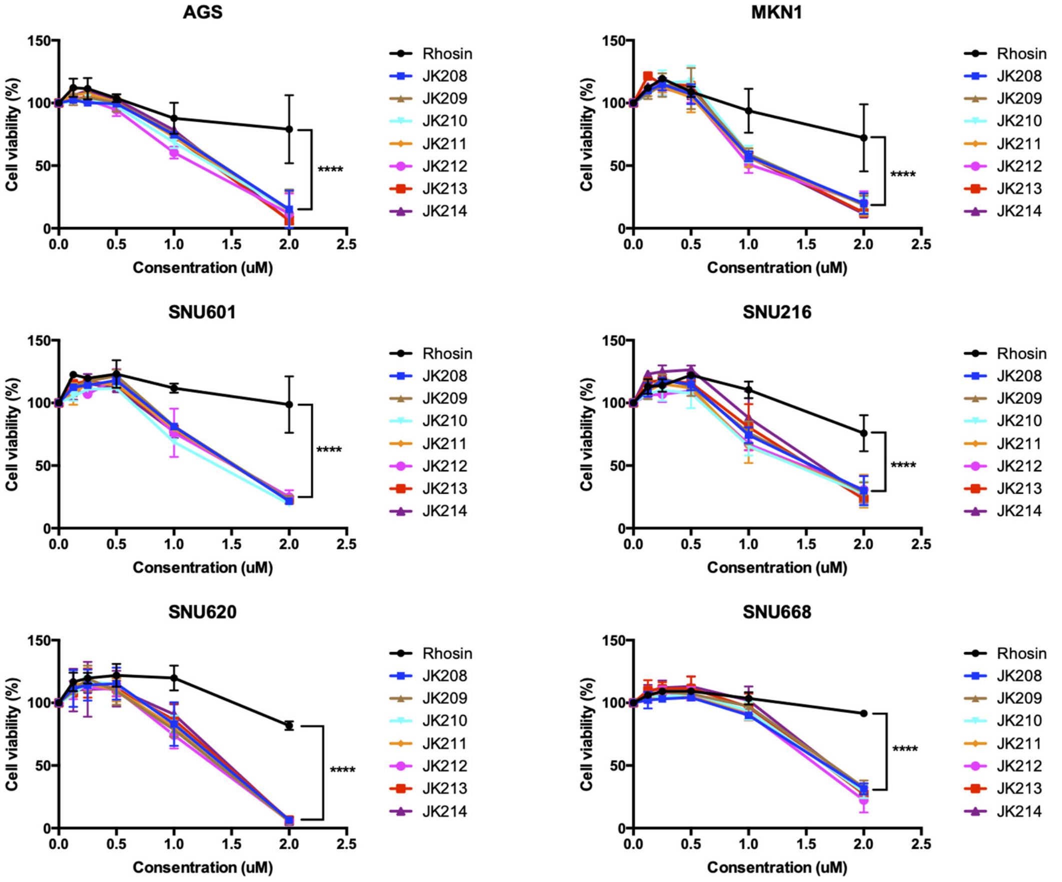

Cell viability assay
(Rhosin, JK-301 ~ JK-307)

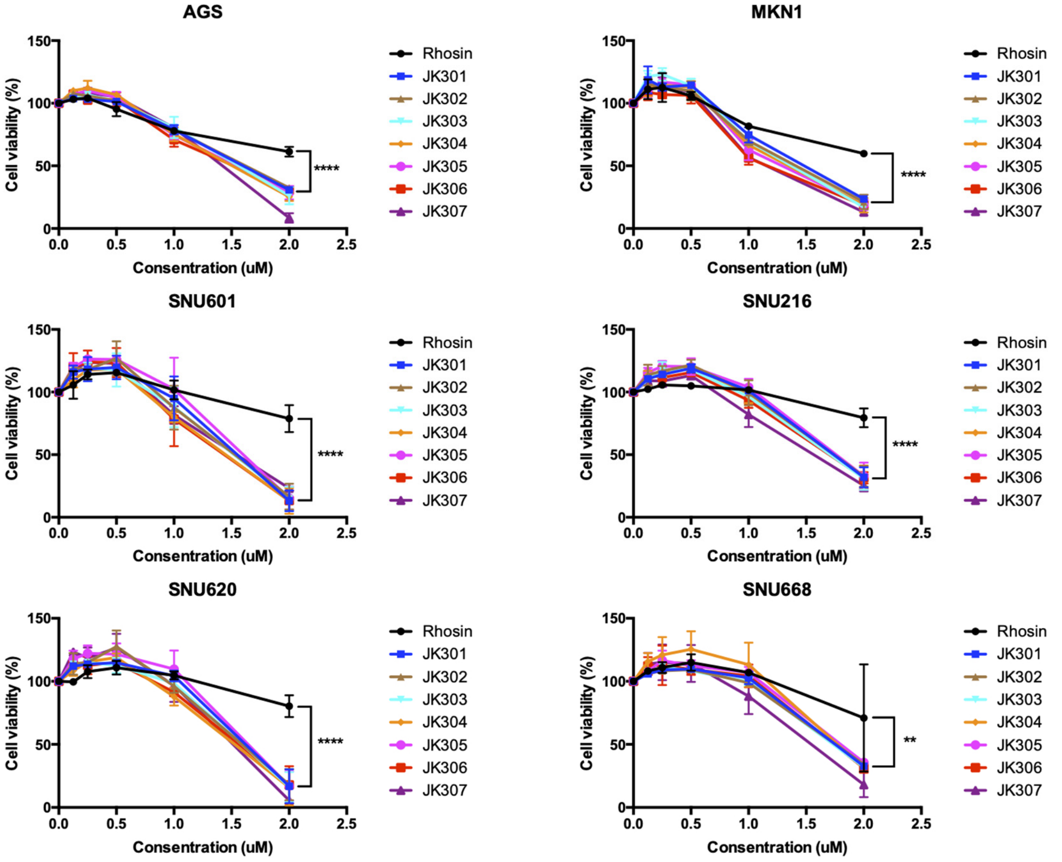

Figure S1. RHOA inhibitors suppress cell growth in GC. GC cell lines, AGS, MKN-1, SNU601, SNU216, SNU620, and SNU668 were treated with the 15 small molecule candidates included Rhosin (** p value < 0.01, **** p value < 0.0001).

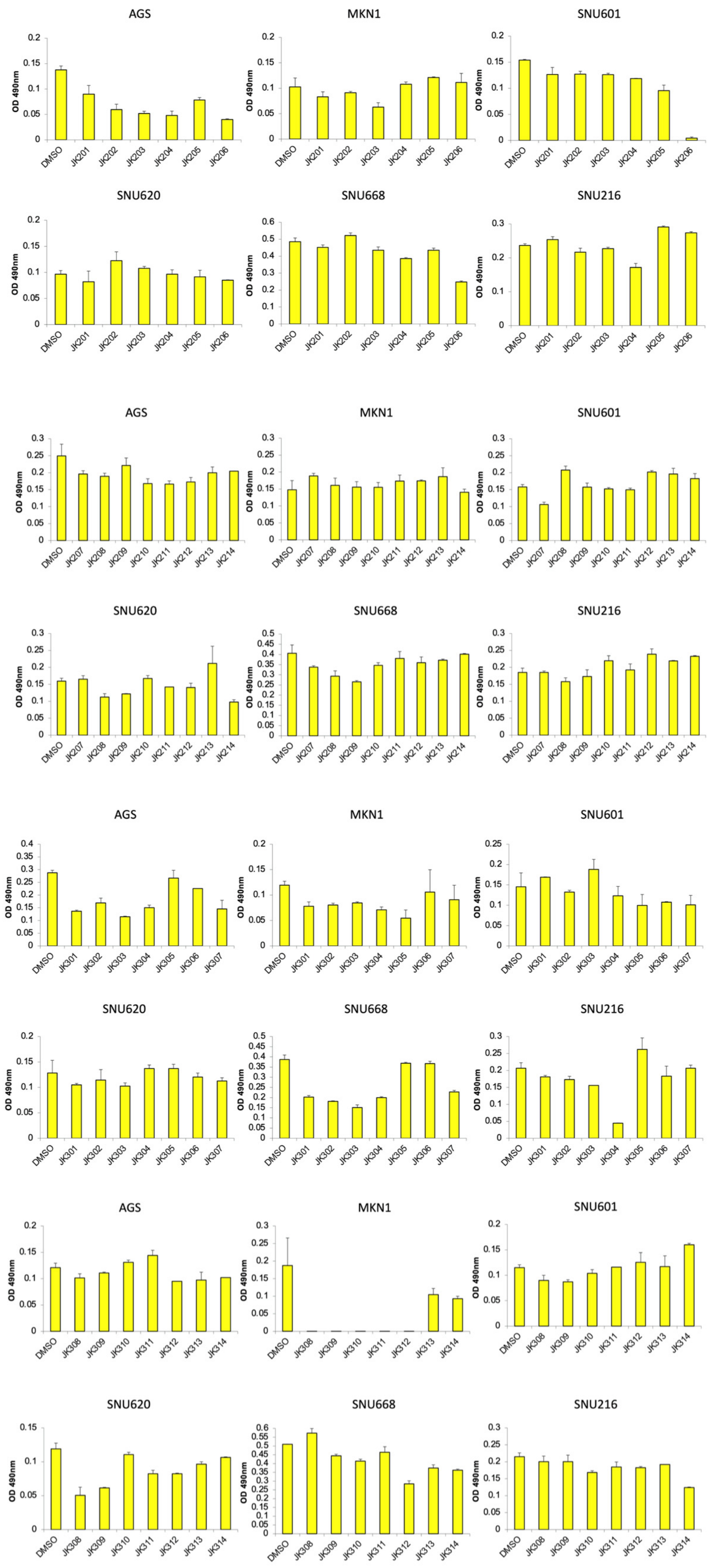

Figure S2. Cell viability assay at 2 μM treatment.

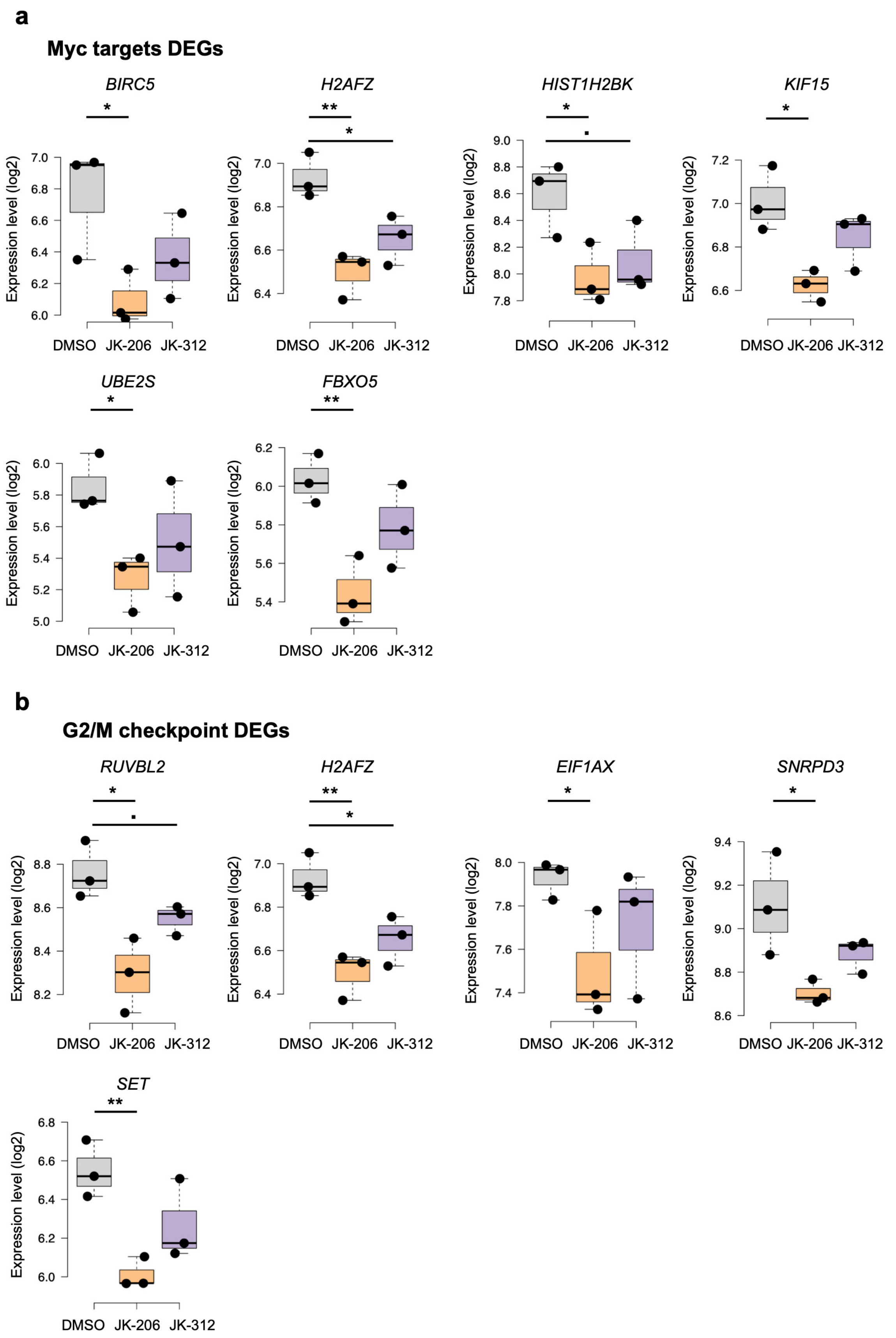

Figure S3. Expression patterns of the DEGs (depicted in Figure 4b) in JK-206, JK-312 and DMSO treatments in GC cells. (**a**) Gene expression profiles involved in the gene set of Myc targets. (**b**)

Gene expression profiles involved in the gene set of G2/M checkpoint. p value < 0.1; *, p value < 0.05; **, p value < 0.01.

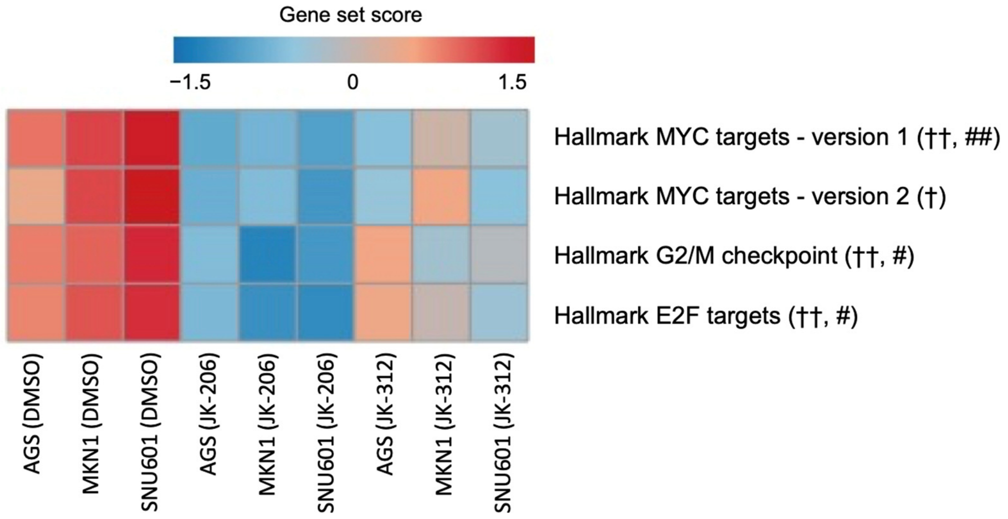

Figure S4. JK-206 and JK-312 treatments in GC cells down-regulated hallmark gene sets of Myc targets, G2/M checkpoint, and E2F targets compared to the DMSO treatment in GC cells. †, *p* value < 0.05; ††, *p* value < 0.01; JK-206 treated vs. DMSO treated GC cell lines; #, *p* value < 0.05; ##, *p* value < 0.01; JK-312 treated vs. DMSO treated GC cell lines (T test).

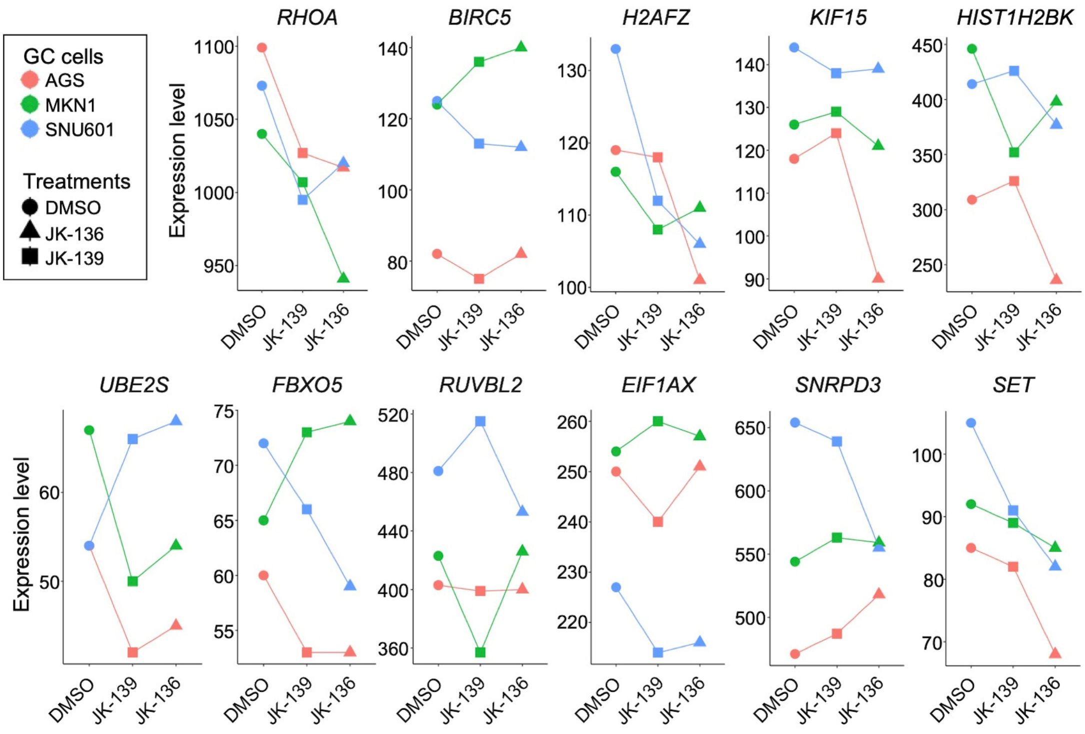

Figure S5. Expression patterns of the DEGs (depicted in Figure 4b) by another RHOA inhibitors in GC cells. Expression levels of *RHOA*, MYC target genes, G2/M checkpoint-related genes in JK-136-, JK-139-, and DMSO-treated GC cells. The data set was obtained from Gene Expression Omnibus (GEO) accession GSE135068 [1].

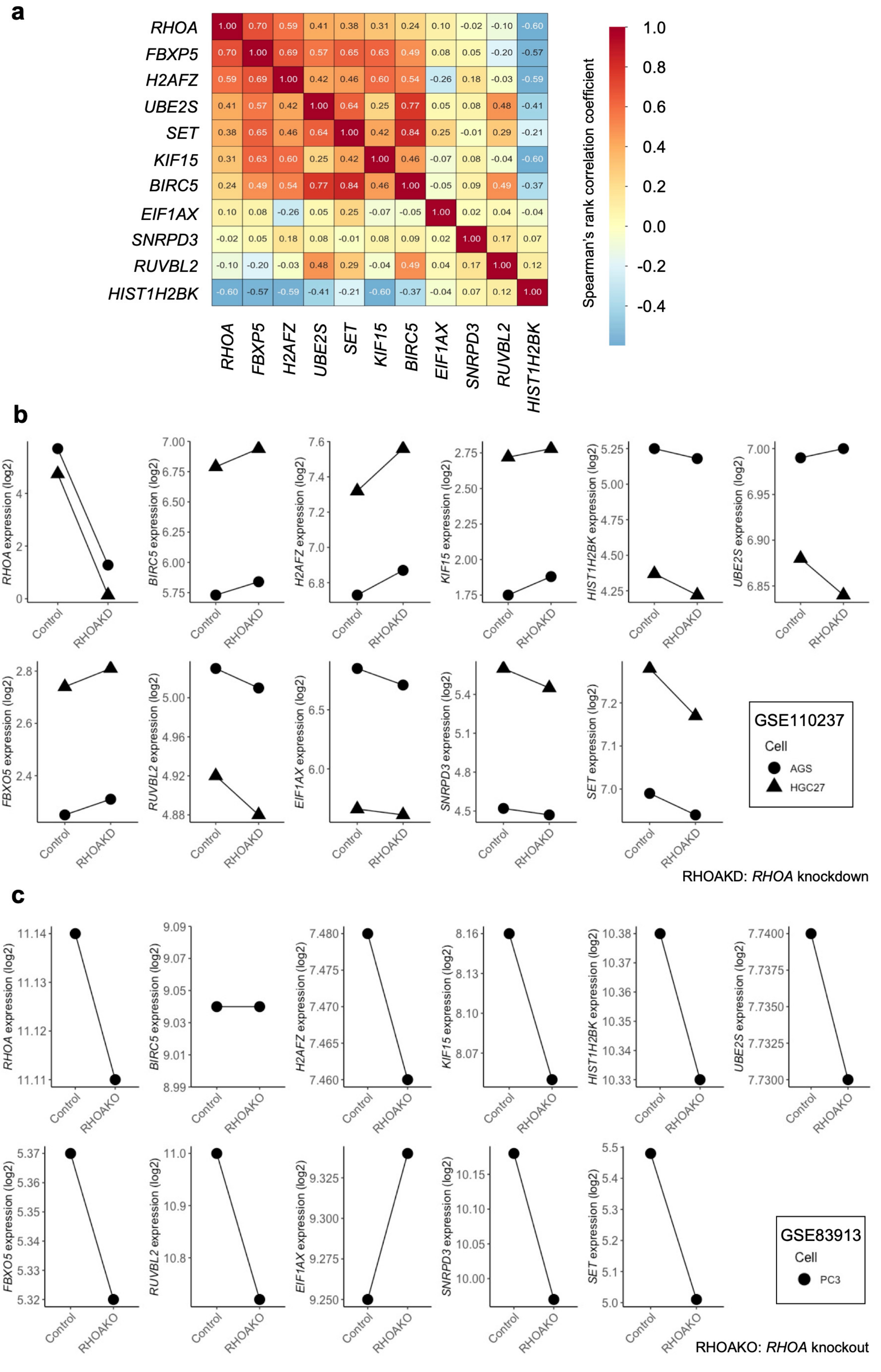

Figure S6. Correlations with *RHOA* and DEGs (depicted in Figure 4b) and expression patterns of the DEGs by knockdown and knockout of *RHOA* in GC and prostate cancer cells. (**a**) Heatmap of correlation coefficients of the ten DEGs (depicted in Figure 4b) with *RHOA* in 13 GC cell lines from

Cancer Cell Line Encyclopedia (CCLE) database [2]. (**b**) MRNA expression levels of *RHOA*, Myc target genes, G2/M checkpoint-related genes in GC cell lines (AGS and HGC27). The dataset was obtained from GEO accession GSE110237 [3]. (**c**) Expression levels of *RHOA*, Myc target genes, G2/M checkpoint-related genes in prostate cell line (PC3). The dataset was obtained from GEO accession GSE83913 [4].

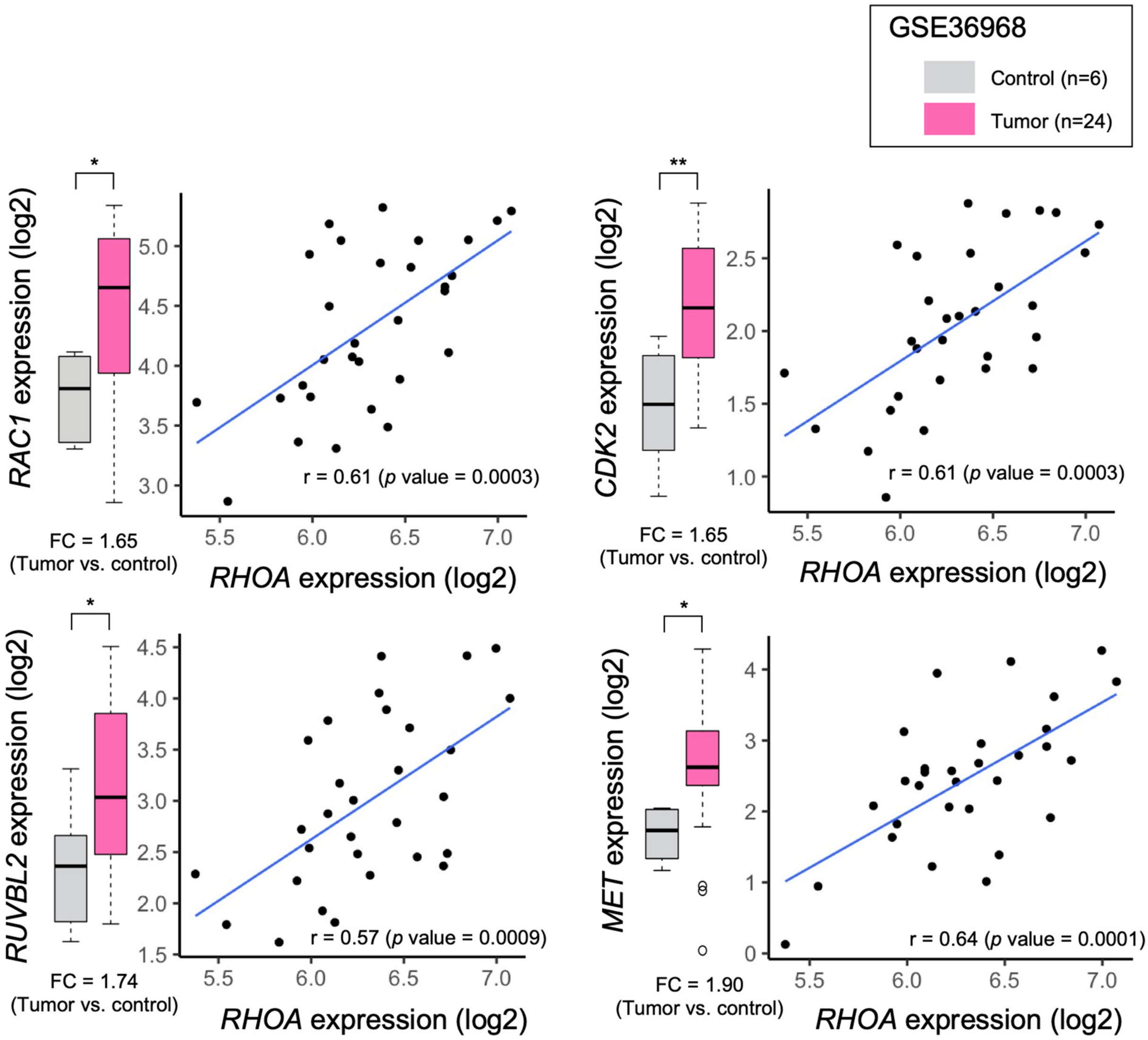

Figure S7. Differentially expressed genes in association with *RHOA* expression level in an independent GC dataset (GSE36968). Analyses of differentially expressed genes and Pearson's correlation coefficients were performed with 20 genes (excluding *RHOA*) from the network (Figure 5a) using the independent GC dataset (GSE36968). r: Pearson's correlation coefficient. Significance: *, p value < 0.05； **, p value < 0.01.

Supplementary Method S1

Synthesis of hydrazide derivatives

(E)-N'-(4-phenoxybenzylidene)benzenesulfonohydrazide (JK-201): a white solid; Yield: 78%; IR (ATR) cm^{-1} 3207, 1589, 1509, 1486, 1448; ^{1}H NMR (400 MHz, DMSO-*d6*) δ 11.46 (s, 1H), 7.89 (m, 3H), 7.61 (m, 5H), 7.41 (t, *J* = 8.0 Hz, 2H), 7.18 (t, *J* = 8.0 Hz, 1H), 7.04 (d, *J* = 8.0 Hz, 2H), 6.98 (d, *J* = 8.0 Hz, 2H); ^{13}C NMR (100 MHz, DMSO-*d6*) δ 158.45, 155.72, 146.58, 139.02, 135.47, 132.99, 130.16, 129.21, 128.66, 127.14, 124.10, 119.27, 118.25; HRMS (ESI) calcd for $C_{19}H_{16}N_2O_3S$ $[M+H]^+$ 353.0960; found 353.0956.

(E)-4-hydroxy-N'-(4-phenoxybenzylidene)benzohydrazide (JK-202): a white solid; Yield: 90%; IR (ATR) cm^{-1} 3171, 3027, 1642, 1604, 1585, 1561, 1502, 1486; ^{1}H NMR (400 MHz, DMSO-*d6*) δ 11.60 (s, 1H), 10.12 (s, 1H), 8.42 (s, 1H), 7.81 (d, *J* = 8.0 Hz, 2H), 7.72 (d, *J* = 8.0 Hz, 2H), 7.43 (t, *J* = 8.0 Hz, 2H), 7.20 (t, *J* = 8.0 Hz, 1H), 7.09 (d, *J* = 8.0 Hz, 2H), 7.05 (d, *J* = 8.0 Hz, 2H) , 6.86 (d, *J* = 8.0 Hz, 2H); ^{13}C NMR (100 MHz, DMSO-*d6*) δ 162.63, 160.63, 158.33, 155.80, 146.19, 130.19, 129.62, 129.55, 128.80, 124.10, 123.90, 119.34, 118.28, 114.99; HRMS (ESI) calcd for $C_{20}H_{16}N_2O_3$ $[M+H]^+$ 333.1239; found 333.1248.

(E)-4-methoxy-N'-(4-phenoxybenzylidene)benzohydrazide (JK-203): a white solid; Yield: 79%; IR (ATR) cm^{-1} 3201, 3016, 1631, 1604, 1544, 1500; ^{1}H NMR (400 MHz, DMSO-*d6*) δ 11.69 (s, 1H), 8.43 (s, 1H), 7.91 (d, *J* = 8.0 Hz, 2H), 7.73 (d, *J* = 8.0 Hz, 2H), 7.43 (t, *J* = 8.0 Hz, 2H), 7.20 (t, *J* = 8.0 Hz, 1H), 7.07(m, 6H), 3.83 (s, 3H); ^{13}C NMR (100 MHz, DMSO-*d6*) δ 162.42, 161.96, 158.40, 155.78, 146.52, 130.20, 129.47, 128.86, 125.47, 124.12, 119.36, 118.27, 113.69, 55.41; HRMS (ESI) calcd for $C_{21}H_{18}N_2O_3$ $[M+H]^+$ 347.1396; found 347.1403.

(E)-4-fluoro-N'-(4-phenoxybenzylidene)benzohydrazide (JK-204): a white solid; Yield: 75%; IR (ATR) cm^{-1} 3235, 3044, 1644, 1599, 1588, 1550, 1500, 1485; ^{1}H NMR (400 MHz, DMSO-*d6*) δ 11.83 (s, 1H), 8.43 (s, 1H), 7.99 (m, 2H), 7.75 (d, *J* = 8.0 Hz, 2H), 7.44 (t, *J* = 8.0 Hz, 2H), 7.37 (t, *J* = 8.0 Hz, 2H), 7.20 (t, *J* = 8.0 Hz, 1H), 7.09 (d, *J* = 8.0 Hz, 2H), 7.06 (d, *J* = 8.0 Hz, 2H); ^{13}C NMR

(100 MHz, DMSO-d_6) δ 164.10 (d, J = 247.0 Hz), 161.94, 158.57, 155.73, 147.24, 130.29 (d, J = 9.0 Hz), 130.21, 129.89, 129.25, 128.99, 124.17, 119.41, 118.24, 115.44 (d, J = 21.0 Hz); HRMS (ESI) calcd for $C_{20}H_{15}FN_2O_2$ $[M+H]^+$ 335.1196; found 335.1205.

(E)-3-nitro-N'-(4-phenoxybenzylidene)benzohydrazide (JK-205): a white solid; Yield: 89%; IR (ATR) cm^{-1} 3186, 3031, 1645, 1600, 1554, 1529, 1504, 1488; ^1H NMR (400 MHz, DMSO-d_6) δ 12.12 (s, 1H), 8.76 (s, 1H), 8.47 (s, 1H), 8.44 (d, J = 8.0 Hz, 1H), 8.37 (d, J = 8.0 Hz, 1H), 7.84 (t, J = 8.0 Hz, 1H), 7.77 (d, J = 8.0, 2H), 7.44 (t, J = 8.0 Hz, 2H), 7.21 (t, J = 8.0 Hz, 1H), 7.10 (d, J = 8.0 Hz, 2H), 7.07 (d, J = 8.0 Hz, 2H); ^{13}C NMR (100 MHz, DMSO-d_6) δ 160.84, 158.77, 155.66, 148.19, 147.75, 134.80, 134.11, 130.29, 130.22, 129.16, 129.01, 126.30, 124.22, 122.26, 119.45, 118.22; HRMS (ESI) calcd for $C_{20}H_{15}N_3O_4$ $[M+H]^+$ 362.1141; found 362.1140.

(E)-N'-(4-phenoxybenzylidene)benzo[d][1,3]dioxole-5-carbohydrazide (JK-206): a white solid; Yield: 96%; IR (ATR) cm^{-1} 3299, 1646, 1605, 1586, 1530, 1483; ^1H NMR (400 MHz, DMSO-d_6) δ 11.66 (s, 1H), 8.42 (s, 1H), 7.73 (d, J = 8.0 Hz, 2H), 7.53 (d, J = 8.0 Hz, 1H), 7.44 (m, 3H), 7.20 (t, J = 8.0 Hz, 1H), 7.07 (m, 5H), 6.13 (s, 2H); ^{13}C NMR (100 MHz, DMSO-d_6) δ 162.07, 158.45, 155.76, 150.12, 147.40, 146.75, 130.20, 129.38, 128.89, 127.21, 124.14, 122.76, 119.38, 118.25, 108.03, 107.56, 101.81; HRMS (ESI) calcd for $C_{21}H_{16}N_2O_4$ $[M+H]^+$ 361.1188; found 361.1193.

(E)-N'-(4-phenoxybenzylidene)-[1,1'-biphenyl]-4-carbohydrazide (JK-207): a white solid; Yield: 88%; IR (ATR) cm^{-1} 3208, 3173, 3035, 1647, 1604, 1585, 1550, 1482; ^1H NMR (400 MHz, DMSO-d_6) δ 11.88 (s, 1H), 8.48 (s, 1H), 8.03 (d, J = 8.0 Hz, 2H), 7.84 (d, J = 8.0 Hz, 2H), 7.76 (d, J = 8.0 Hz, 4H), 7.51 (t, J = 8.0 Hz, 2H), 7.44 (m, 3H), 7.21 (t, J = 8.0 Hz, 1H), 7.08 (m, 4H); ^{13}C NMR (100 MHz, DMSO-d_6) δ 162.62, 158.54, 155.73, 147.14, 143.23, 139.06, 132.18, 130.21, 129.33, 129.04, 128.98, 128.29, 128.15, 126.90, 126.66, 124.17, 119.41, 118.24; HRMS (ESI) calcd for $C_{26}H_{20}N_2O_2$ $[M+H]^+$ 393.1603; found 393.1615.

(E)-N'-(4-phenoxybenzylidene)furan-2-carbohydrazide (JK-208): a white solid; Yield: 76%; IR (ATR) cm^{-1} 3164, 3016, 2998, 1641, 1585, 1542, 1503, 1474; ^1H NMR (400 MHz, DMSO-d_6) δ 11.80 (s, 1H), 8.43 (s, 1H), 7.94 (s, 1H), 7.72 (d, J = 8.0 Hz, 2H), 7.43 (t, J = 8.0 Hz, 2H), 7.29 (s, 1H),

7.20 (t, *J* = 8.0 Hz, 1H), 7.09 (d, *J* = 8.0 Hz, 2H), 7.05 (d, *J* = 8.0 Hz, 2H), 6.70 (s, 1H); ^{13}C NMR (100 MHz, DMSO-*d6*) δ 158.54, 155.73, 154.11, 147.21, 146.66, 145.77, 130.20, 129.22, 128.97, 124.15, 119.39, 118.25, 114.82, 112.06; HRMS (ESI) calcd for $C_{18}H_{14}N_2O_3$ [M+H]$^+$ 307.1083; found 307.1087.

(E)-N'-(4-phenoxybenzylidene)isonicotinohydrazide (JK-209): a pale yellow solid; Yield: 80%; IR (ATR) cm^{-1} 3236, 3065, 1653, 1587, 1551, 1487; ^{1}H NMR (400 MHz, DMSO-*d6*) δ 12.03 (s, 1H), 8.79 (d, *J* = 4.0 Hz, 2H), 8.45 (s, 1H), 7.82 (d, *J* = 4.0 Hz, 2H), 7.77 (d, *J* = 8.0 Hz, 2H), 7.44 (t, *J* = 8.0 Hz, 2H), 7.21 (t, *J* = 8.0 Hz, 1H), 7.08 (m, 4H); ^{13}C NMR (100 MHz, DMSO-*d6*) δ 161.54, 158.86, 155.67, 150.34, 148.44, 140.53, 130.26, 129.23, 128.97, 124.28, 121.54, 119.51, 118.24; HRMS (ESI) calcd for $C_{19}H_{15}N_3O_2$ [M+Na]$^+$ 340.1062; found 340.1056.

(E)-N'-(4-phenoxybenzylidene)imidazo[1,2-a]pyridine-7-carbohydrazide (JK-210): an off-white solid; Yield: 82%; IR (ATR) cm^{-1} 3201, 3043, 1670, 1630, 1586, 1549, 1504, 1485; ^{1}H NMR (400 MHz, DMSO-*d6*) δ 11.98 (s, 1H), 8.67 (d, *J* = 8.0 Hz, 1H), 8.47 (s, 1H), 8.27 (s, 1H), 8.11 (s, 1H), 7.77 (m, 3H), 7.44 (t, *J* = 8.0 Hz, 2H), 7.38 (d, *J* = 8.0 Hz, 1H), 7.20 (t, *J* = 8.0 Hz, 1H), 7.08 (m, 4H); ^{13}C NMR (100 MHz, DMSO-*d6*) δ 161.41, 158.63, 155.70, 147.49, 143.37, 135.25, 130.20, 129.19, 129.05, 128.61, 126.93, 124.17, 119.42, 118.23, 116.35, 114.47, 110.51; HRMS (ESI) calcd for $C_{21}H_{16}N_4O_2$ [M+H]$^+$ 357.1352; found 357.1359.

(E)-N'-(4-phenoxybenzylidene)-2-phenylacetohydrazide (JK-211): an off-white solid; Yield: 84%; IR (ATR) cm^{-1} 3210, 3055, 1667, 1586, 1555, 1504, 1486; ^{1}H NMR (400 MHz, DMSO-*d6*) δ 11.56 (s, 0.4H), 11.35 (s, 0.6H), 8.20 (s, 0.4H), 7.98 (s, 0.6H), 7.70 (m, 2H), 7.31 (m, 8H), 7.05 (m, 4H), 3.97 (s, 1.2H), 3.53 (s, 0.8H); ^{13}C NMR (100 MHz, DMSO-*d6*) δ 172.15, 166.40, 158.43, 158.16, 155.85, 155.73, 145.89, 142.21, 135.74, 135.70, 130.17, 129.36, 129.04, 128.87, 128.57, 128.29, 128.20, 126.55, 126.33, 124.12, 124.05, 119.36, 119.23, 118.38, 118.17, 41.22; HRMS (ESI) calcd for $C_{21}H_{18}N_2O_2$ [M+H]$^+$ 331.1447; found 331.1453.

(E)-2-(4-fluorophenoxy)-N'-(4-phenoxybenzylidene)acetohydrazide (JK-212): a white solid; Yield: 91%; IR (ATR) cm^{-1} 3213, 3071, 1691, 1607, 1587, 1504, 1488; ^{1}H NMR (400 MHz, DMSO-*d6*) δ 11.54 (s, 1H), 8.32 (s, 0.4H), 7.99 (s, 0.6H), 7.71 (m, 2H), 7.43 (m, 2H), 7.10 (m, 9H), 5.11 (s, 1.2H), 4.64 (s, 0.8H); ^{13}C NMR (100 MHz, DMSO-*d6*) δ 168.83, 164.00, 158.63, 158.29, 158.04, 157.73, 155.84, 155.68, 155.38, 154.51, 154.06, 147.32, 143.15, 130.20, 130.18, 129.02, 128.80,

124.18, 124.06, 119.42, 119.19, 118.32, 118.18, 116.10, 116.02, 115.98, 115.81, 115.75, 115.73, 115.57, 67.03, 65.22; HRMS (ESI) calcd for $C_{21}H_{17}FN_2O_3$ [M+H]$^+$ 365.1301; found 365.1294.

(E)-2-((1H-benzo[d]imidazol-2-yl)thio)-N'-(4-phenoxybenzylidene)acetohydrazide (JK-213): a white solid; Yield: 77%; IR (ATR) cm^{-1} 3206, 3150, 3051, 1667, 1587, 1485; ^{1}H NMR (400 MHz, DMSO-*d6*) δ 12.61 (s, 1H), 11.61 (s, 1H), 8.19 (s, 0.4H), 8.01 (s, 0.6H), 7.69 (m, 2H), 7.43 (t, *J* = 8.0Hz, 4H), 7.20 (t, *J* = 8.0Hz, 1H), 7.08 (m, 6H), 4.58 (s, 1.2H), 4.18 (s, 0.8H); ^{13}C NMR (100 MHz, DMSO-*d6*) δ 168.98, 163.79, 158.59, 158.28, 155.80, 155.69, 149.81, 149.60, 146.37, 142.94, 130.20, 130.18, 129.05, 129.01, 128.74, 124.17, 124.08, 121.38, 119.42, 119.24, 118.30, 118.16, 34.25, 33.60; HRMS (ESI) calcd for $C_{22}H_{18}N_4O_2S$ [M+H]$^+$ 403.1229; found 403.1235.

(E)-1-benzyl-N'-(4-phenoxybenzylidene)pyrrolidine-3-carbohydrazide (JK-214): a white solid; Yield: 86%; IR (ATR) cm^{-1} 3168, 3015, 1668, 1648, 1586, 1561, 1487; ^{1}H NMR (400 MHz, DMSO-*d6*) δ 11.28 (s, 0.4H), 11.23 (s, 0.6H), 8.13 (s, 0.4H), 7.94 (s, 0.6H), 7.65 (m, 2H), 7.42 (t, *J* = 8.0Hz, 2H), 7.31 (m, 4H), 7.21 (m, 2H), 7.00-7.08 (m, 4H), 3.64 (m, 3H), 2.87 (m, 1H), 2.42-2.68 (m, 3H), 1.99 (m, 2H); ^{13}C NMR (100 MHz, DMSO-*d6*) δ 175.21, 170.00, 158.34, 158.01, 155.90, 155.75, 145.41, 141.78, 139.10, 130.18, 130.15, 129.45, 129.31, 128.79, 128.51, 128.46, 128.44, 128.13, 128.10, 126.81, 126.75, 124.11, 124.00, 119.35, 119.15, 118.41, 118.19, 59.25, 59.16, 56.89, 56.36, 53.54, 41.47, 27.45, 26.96; HRMS (ESI) calcd for $C_{25}H_{25}N_3O_2$ [M+Na]$^+$ 422.1844; found 422.1844.

(E)-N'-((6-(benzo[d][1,3]dioxol-5-yl)pyridin-2-yl)methylene)benzenesulfonohydrazide (JK-301): a yellow solid; Yield: 78%; IR (ATR) cm^{-1} 3229, 1588, 1566, 1504, 1459; ^{1}H NMR (400 MHz, DMSO-*d6*) δ 11.92 (s, 1H), 7.97 (s, 1H), 7.83-7.92 (m, 4H), 7.61-7.68 (m, 6H), 7.02 (d, *J* = 12.0 Hz, 1H), 6.08 (s, 2H); ^{13}C NMR (100 MHz, DMSO-*d6*) δ 155.39, 152.02, 148.34, 147.94, 147.34, 138.85, 137.93, 133.22, 132.24, 129.36, 127.12, 120.81, 120.25, 117.66, 108.46, 106.61, 101.38; HRMS (ESI) calcd for $C_{19}H_{15}N_3O_4S$ [M+Na]$^+$ 404.0681; found 404.0691.

(E)-N'-((6-(benzo[d][1,3]dioxol-5-yl)pyridin-2-yl)methylene)-4-hydroxybenzohydrazide (JK-302): a white solid; Yield: 95%; IR (ATR) cm^{-1} 3066, 1656, 1605, 1584, 1542, 1504; ^{1}H NMR (400 MHz, DMSO-*d6*) δ 11.87 (s, 1H), 10.18 (s, 1H), 8.52 (s, 1H), 7.84-7.90 (m, 5H), 7.67 (s, 2H), 7.04 (d, *J* = 8.0 Hz, 1H), 6.89 (d, *J* = 8.0 Hz, 2H), 6.10 (s, 2H); ^{13}C NMR (100 MHz, DMSO-*d6*) δ 160.85, 155.37, 153.14, 148.31, 147.97, 147.30, 137.80, 132.47, 129.82, 123.61, 120.79, 119.98, 117.93, 115.08, 108.49, 106.63, 101.39; HRMS (ESI) calcd for $C_{20}H_{15}N_3O_4$ [M+Na]$^+$ 384.0960; found 384.0953.

(E)-N'-((6-(benzo[d][1,3]dioxol-5-yl)pyridin-2-yl)methylene)-4-methoxybenzohydrazide (JK-303): a white solid; Yield: 98%; IR (ATR) cm^{-1} 3327, 3204, 1645, 1603, 1543, 1505; ^{1}H NMR (400 MHz, DMSO-*d6*) δ 11.94 (s, 1H), 8.54 (s, 1H), 7.87-7.95 (m, 5H), 7.68 (s, 2H), 7.04-7.10 (m, 3H), 6.10 (s, 2H), 3.85 (s, 3H); ^{13}C NMR (100 MHz, DMSO-*d6*) δ 162.16, 155.38, 153.05, 148.32, 147.97, 147.61, 137.85, 132.45, 129.67, 125.17, 120.80, 120.05, 117.97, 113.80, 108.50, 106.62, 101.39, 55.46; HRMS (ESI) calcd for $C_{21}H_{17}N_3O_4$ $[M+Na]^+$ 398.1117; found 398.1110.

(E)-N'-((6-(benzo[d][1,3]dioxol-5-yl)pyridin-2-yl)methylene)-4-fluorobenzohydrazide (JK-304): a white solid; Yield: 86%; IR (ATR) cm^{-1} 3265, 3070, 1648, 1599, 1547, 1501; ^{1}H NMR (400 MHz, DMSO-*d6*) δ 12.09 (s, 1H), 8.54 (s, 1H), 7.89-8.03 (m, 5H), 7.67 (m, 2H), 7.40 (t, *J* = 8.0 Hz, 2H), 7.04 (d, *J* = 8.0 Hz, 1H), 6.10 (s, 2H); ^{13}C NMR (100 MHz, DMSO-*d6*) δ 164.25 (d, *J* = 247.0 Hz), 162.25, 155.42, 152.88, 148.33, 147.97, 139.36, 137.85, 132.40, 130.47 (d, *J* = 8.0 Hz), 129.64, 120.80, 120.20, 118.07, 115.56 (d, *J* = 22.0 Hz), 108.48, 106.62, 101.39; HRMS (ESI) calcd for $C_{20}H_{14}FN_3O_3$ $[M+Na]^+$ 386.0917; found 386.0913.

(E)-N'-((6-(benzo[d][1,3]dioxol-5-yl)pyridin-2-yl)methylene)-3-nitrobenzohydrazide (JK-305): a pale yellow solid; Yield: 93%; IR (ATR) cm^{-1} 3193, 3051, 1651, 1530, 1501; ^{1}H NMR (400 MHz, DMSO-*d6*) δ 12.36 (s, 1H), 8.79 (s, 1H), 8.57 (s, 1H), 8.46 (d, *J* = 8.0 Hz, 1H), 8.40 (d, *J* = 8.0 Hz, 1H), 7.84-7.93 (m, 4H), 7.68 (m, 2H), 7.04 (d, *J* = 8.0 Hz, 1H), 6.10 (s, 2H); ^{13}C NMR (100 MHz, DMSO-*d6*) δ 161.17, 155.46, 152.66, 149.29, 148.36, 147.97, 147.79, 137.91, 134.50, 134.21, 132.35, 130.38, 126.53, 122.39, 120.80, 120.37, 118.21, 108.49, 106.61, 101.40; HRMS (ESI) calcd for $C_{20}H_{14}N_4O_5$ $[M+H]^+$ 391.1042; found 391.1050.

(E)-N'-((6-(benzo[d][1,3]dioxol-5-yl)pyridin-2-yl)methylene)benzo[d][1,3]dioxole-5-carbohydrazide (JK-306): an off-white solid; Yield: 96%; IR (ATR) cm^{-1} 3506, 3189, 1649, 1603, 1557, 1501, 1487; ^{1}H NMR (400 MHz, DMSO-*d6*) δ 11.91 (s, 1H), 8.52 (s, 1H), 7.87-7.92 (m, 3H), 7.68 (m, 2H), 7.56 (d, *J* = 8.0 Hz, 1H), 7.48 (s, 1H), 7.08 (d, *J* = 8.0 Hz, 1H), 7.05 (d, *J* = 8.0 Hz, 1H), 6.15 (s, 2H), 6.10 (s, 2H); ^{13}C NMR (100 MHz, DMSO-*d6*) δ 162.36, 155.40, 152.98, 150.35, 148.33, 147.97, 147.83, 147.46, 137.86, 132.43, 126.91, 122.99, 120.80, 120.10, 118.00, 108.50, 108.11, 107.67, 106.62, 101.88, 101.39; HRMS (ESI) calcd for $C_{21}H_{15}N_3O_5$ $[M+H]^+$ 390.1090; found

摫

(E)-N'-((6-(benzo[d][1,3]dioxol-5-yl)pyridin-2-yl)methylene)-[1,1'-biphenyl]-4-carbohydrazide (JK-307): a white solid; Yield: 93%; IR (ATR) cm^{-1} 3221, 3051, 1651, 1541, 1501; ^{1}H NMR (400

MHz, DMSO-d_6) δ 12.13 (s, 1H), 8.58 (s, 1H), 8.06 (d, J = 8.0 Hz, 2H), 7.85-7.92 (m, 5H), 7.77 (d, J = 8.0 Hz, 2H), 7.69 (s, 2H), 7.51 (t, J = 8.0 Hz, 2H), 7.43 (t, J = 8.0 Hz, 1H), 7.05 (d, J = 8.0 Hz, 1H), 6.10 (s, 2H); ^{13}C NMR (100 MHz, DMSO-d_6) δ 162.95, 155.42, 152.96, 148.33, 148.23, 147.98, 143.48, 139.01, 137.86, 132.42, 131.89, 129.06, 128.42, 128.21, 126.93, 126.74, 120.81, 120.16, 118.07, 108.50, 106.63, 101.39; HRMS (ESI) calcd for $C_{26}H_{19}N_3O_3$ [M+H]$^+$ 422.1505; found 422.1521.

(E)-N'-((6-(benzo[d][1,3]dioxol-5-yl)pyridin-2-yl)methylene)furan-2-carbohydrazide (JK-308): a white solid; Yield: 90%; IR (ATR) cm^{-1} 3216, 3052, 1651, 1585, 1563, 1547, 1504; ^{1}H NMR (400 MHz, DMSO-d_6) δ 12.09 (s, 1H), 8.54 (s, 1H), 7.86-7.99 (m, 4H), 7.68 (s, 2H), 7.35 (s, 1H), 7.05 (d, J = 8.0 Hz, 1H), 6.74 (s, 1H), 6.10 (s, 2H); ^{13}C NMR (100 MHz, DMSO-d_6) δ 155.44, 154.28, 152.85, 148.34, 148.25, 147.98, 146.37, 146.13, 137.90, 132.41, 120.83, 120.18, 118.14, 115.44, 112.19, 108.51, 106.64, 101.40; HRMS (ESI) calcd for $C_{18}H_{13}N_3O_4$ [M+H]$^+$ 336.0984; found 336.0986.

(E)-N'-((6-(benzo[d][1,3]dioxol-5-yl)pyridin-2-yl)methylene)isonicotinohydrazide (JK-309): a white solid; Yield: 86%; IR (ATR) cm^{-1} 3235, 3074, 1654, 1545, 1500, 1453; ^{1}H NMR (400 MHz, DMSO-d_6) δ 12.28 (s, 1H), 8.82 (d, J = 4.0 Hz, 2H), 8.55 (s, 1H), 7.82-7.93 (m, 5H), 7.68 (m, 2H), 7.05 (d, J = 8.0 Hz, 1H), 6.10 (s, 2H); ^{13}C NMR (100 MHz, DMSO-d_6) δ 161.88, 155.50, 152.62, 150.40, 149.43, 148.36, 147.98, 140.26, 137.95, 132.35, 121.56, 120.83, 120.44, 118.25, 108.51, 106.63, 101.40; HRMS (ESI) calcd for $C_{19}H_{14}N_4O_3$ [M+H]$^+$ 347.1144; found 347.1156.

(E)-N'-((6-(benzo[d][1,3]dioxol-5-yl)pyridin-2-yl)methylene)imidazo[1,2-a]pyridine-7-carbohydrazide (JK-310): an off-white solid; Yield: 93%; IR (ATR) cm^{-1} 2964, 1655, 1609, 1535, 1518, 1503; ^{1}H NMR (400 MHz, DMSO-d_6) δ 12.22 (s, 1H), 8.69 (d, J = 8.0 Hz, 1H), 8.57 (s, 1H), 8.31 (s, 1H), 8.13 (s, 1H), 7.91 (m, 3H), 7.79 (s, 1H), 7.68 (m, 2H), 7.40 (d, J = 4.0 Hz, 1H), 7.04 (d, J = 8.0 Hz, 1H), 6.10 (s, 2H); ^{13}C NMR (100 MHz, DMSO-d_6) δ 161.74, 155.44, 152.83, 148.56, 148.34, 147.97, 143.34, 137.85, 135.39, 132.39, 128.26, 127.02, 120.81, 120.22, 118.12, 116.65, 114.57, 110.48, 108.48, 106.63, 101.39; HRMS (ESI) calcd for $C_{21}H_{15}N_5O_3$ [M+H]$^+$ 386.1253; found 386.1258.

(E)-N'-((6-(benzo[d][1,3]dioxol-5-yl)pyridin-2-yl)methylene)-2-phenylacetohydrazide (JK-311): a white solid; Yield: 84%; IR (ATR) cm^{-1} 3202, 3052, 1665, 1542, 1500; ^{1}H NMR (400 MHz, DMSO-d_6) δ 11.85 (s, 0.3H), 11.65 (s, 0.7H), 8.30 (s, 0.3H), 8.09 (s, 0.7H), 7.79-7.89 (m, 3H), 7.66 (m, 2H),

7.23-7.33 (m, 5H), 7.03 (d, *J* = 8.0 Hz, 1H), 6.10 (s, 2H), 4.03 (s, 1.4H), 3.59 (s, 0.6H); ^{13}C NMR (100 MHz, DMSO-*d6*) δ 172.55, 166.84, 155.36, 152.76, 148.31, 147.95, 146.99, 143.50, 137.82, 135.54, 135.41, 132.40, 129.42, 129.10, 128.34, 128.23, 126.64, 126.42, 120.79, 120.10, 119.90, 117.99, 117.69, 108.46, 106.62, 101.38, 41.22; HRMS (ESI) calcd for $C_{21}H_{17}N_3O_3$ [M+H]$^+$ 360.1348; found 360.1358.

(E)-N'-((6-(benzo[d][1,3]dioxol-5-yl)pyridin-2-yl)methylene)-2-(4-fluorophenoxy)acetohydrazide (JK-312): a white solid; Yield: 89%; IR (ATR) cm^{-1} 3069, 1701, 1570, 1501; ^{1}H NMR (400 MHz, DMSO-*d6*) δ 11.83 (s, 1H), 8.43 (s, 0.3H), 8.08 (s, 0.7H), 7.82-7.91 (m, 3H), 7.66 (s, 2H), 6.98-7.19 (m, 5H), 6.10 (s, 2H), 5.18 (s, 1.3H), 4.70 (s, 0.7H); ^{13}C NMR (100 MHz, DMSO-*d6*) δ 169.23, 164.52, 155.43, 15534, 154.48, 154.01, 152.70, 152.48, 148.40, 148.33, 147.96, 144.39, 137.86, 137.75, 132.35, 120.81, 120.27, 120.07, 118.12, 117.91, 116.15, 116.07, 116.00, 115.87, 115.81, 115.78, 115.58, 108.47, 106.62, 101.39, 67.03, 65.22; HRMS (ESI) calcd for $C_{21}H_{16}FN_3O_4$ [M+Na]$^+$ 416.1023; found 416.1014.

(E)-2-((1H-benzo[d]imidazol-2-yl)thio)-N'-((6-(benzo[d][1,3]dioxol-5-yl)pyridin-2-yl)methylene)acetohydrazide (JK-313): an off-white solid; Yield: 84%; IR (ATR) cm^{-1} 3043, 2958, 1676, 1600; ^{1}H NMR (400 MHz, DMSO-*d6*) δ 12.62 (s, 1H), 8.00-8.09 (m, 2H), 7.66-7.80 (m, 2H), 7.54 (m, 3H), 7.37 (s, 1H), 7.04-7.18 (m, 4H), 6.06 (m, 2H), 4.68 (s, 1.5H), 4.27 (s, 0.5H); ^{13}C NMR (100 MHz, DMSO-*d6*) δ 169.59, 164.93, 155.36, 154.90, 151.47, 151.32, 149.65, 149.08, 148.89, 148.30, 148.23, 143.54, 139.40, 138.76, 136.10, 135.49, 131.43, 124.73, 121.65, 121.46, 121.20, 121.09, 117.33, 117.03, 110.39, 110.31, 108.70, 107.28, 106.57, 101.64, 101.53, 34.59, 33.28; HRMS (ESI) calcd for $C_{22}H_{17}N_5O_3S$ [M+H]$^+$ 432.1130; found 432.1138.

(E)-N'-((6-(benzo[d][1,3]dioxol-5-yl)pyridin-2-yl)methylene)-1-benzylpyrrolidine-3-carbohydrazide (JK-314): a white solid; Yield: 77%; IR (ATR) cm^{-1} 3437, 3152, 1670, 1555, 1504; ^{1}H NMR (400 MHz, DMSO-*d6*) δ 11.56 (s, 0.4H), 11.53 (s, 0.6H), 8.23 (s, 0.4H), 8.04 (s, 0.6H), 7.87 (s, 2H), 7.77 (m, 1H), 7.64 (s, 2H), 7.24-7.31 (m, 5H), 7.03 (d, *J* = 8.0 Hz, 1H), 6.09 (s, 2H), 3.71 (m, 1H), 3.59 (m, 2H), 2.91 (m, 1H), 2.62 (m, 2H), 2.44 (m, 1H), 2.04 (m, 2H); ^{13}C NMR (100 MHz, DMSO-*d6*) δ 175.64, 170.44, 155.32, 152.86, 148.29, 147.94, 146.52, 143.14, 139.15, 137.77, 132.41, 128.50, 128.46, 128.11, 126.80, 126.75, 120.78, 120.03, 119.81, 117.93, 117.50, 108.46, 106.61, 101.37, 59.26, 59.14, 56.81, 56.32, 53.52, 41.59, 27.41, 27.05; HRMS (ESI) calcd for $C_{25}H_{24}N_4O_3$ [M+H]$^+$ 429.1927; found 429.1931.

Table S1. A list of DEGs common and uncommon to JK-206 treated (versus DMSO treated) and JK-312 treated (versus DMSO treated) GC cells.

Category	JK-206 treated versus DMSO treated GC cell lines			JK-312 treated versus DMSO treated GC cell lines		
	Symbol	FC	P	Symbol	FC	P
Common DEGs in JK-206 and -312 treated GC cell lines	*ALKAL2*	1.17	0.04	*ALKAL2*	1.16	0.01
	ALYREF	0.57	0.02	*ALYREF*	0.63	<0.05
	B9D2	0.78	0.01	*B9D2*	0.80	0.01
	C5orf49	1.25	0.02	*C5orf49*	1.15	0.03
	CAB39L	1.42	0.02	*CAB39L*	1.40	<0.05
	CACNA1C-IT3	1.30	0.04	*CACNA1C-IT3*	1.24	0.01
	CACTIN	0.93	0.04	*CACTIN*	0.94	0.04
	CCL22	1.33	0.03	*CCL22*	1.20	0.01
	CCT6A	0.70	0.02	*CCT6A*	0.77	<0.05
	CFAP20	0.73	0.01	*CFAP20*	0.82	0.03
	DEFB126	0.87	0.04	*DEFB126*	0.75	<0.01
	DIRC1	0.66	<0.01	*DIRC1*	0.82	0.03
	DNAI3	1.20	<0.05	*DNAI3*	1.25	0.02
	DYTN	1.19	0.04	*DYTN*	1.34	0.02
	FAM66C	0.87	0.04	*FAM66C*	1.24	<0.05
	FAM83D	0.64	0.02	*FAM83D*	0.68	0.03
	FOXM1	0.70	0.02	*FOXM1*	0.75	0.04
	FRZB	0.86	0.04	*FRZB*	0.81	0.03
	GDI2	0.88	<0.01	*GDI2*	0.88	0.03
	GGA2	0.87	0.03	*GGA2*	0.84	0.01
	GPR65	1.28	0.03	*GPR65*	1.24	0.02
	GSTM5	0.78	0.03	*GSTM5*	0.79	<0.05
	H1-5	0.46	0.03	*H1-5*	0.48	0.03
	H2AFZ	0.74	0.01	*H2AFZ*	0.82	0.04
	H4C11	0.74	<0.01	*H4C11*	0.83	0.03
	HCG24	1.24	0.01	*HCG24*	1.13	0.04
	HMGCS2	1.16	0.02	*HMGCS2*	1.11	<0.05
	HMGN1P30	0.71	0.03	*HMGN1P30*	0.81	<0.01
	HNRNPL	0.77	0.02	*HNRNPL*	0.80	0.03
	KPTN	0.89	0.02	*KPTN*	0.84	<0.01
	LIAS	0.80	0.01	*LIAS*	0.85	<0.05
	LINC00482	1.20	0.01	*LINC00482*	1.22	0.01
	LINC00527	1.33	<0.01	*LINC00527*	1.37	0.01
	LINC00534	0.76	0.02	*LINC00534*	0.74	0.02
	LINC00615	0.84	0.03	*LINC00615*	0.73	0.04

	LINC00656	0.76	<0.01	*LINC00656*	0.82	<0.01
	LINC00693	1.16	<0.01	*LINC00693*	1.18	0.02
	LINC01488	0.70	0.01	*LINC01488*	0.66	0.03
	LINC01500	1.28	0.04	*LINC01500*	1.21	0.02
	LNP1	1.35	0.03	*LNP1*	1.25	0.02
	LOC100128908	1.46	0.01	*LOC100128908*	1.29	0.03
	LOC100507291	1.41	<0.05	*LOC100507291*	1.37	0.04
	LOC101927355	0.82	<0.05	*LOC101927355*	0.78	0.03
	LOC101928093	1.16	0.04	*LOC101928093*	1.21	<0.01
	LOC101928596	0.79	0.04	*LOC101928596*	0.77	0.01
	LOC101929646	1.17	0.02	*LOC101929646*	1.26	0.01
	LOC102467222	1.43	0.03	*LOC102467222*	1.45	<0.05
	LOC102723530	1.19	<0.01	*LOC102723530*	1.36	<0.01
	LOC102724152	1.40	0.01	*LOC102724152*	1.14	0.03
	LOC102725168	0.78	0.02	*LOC102725168*	0.81	0.02
	LOC102725254	1.19	<0.05	*LOC102725254*	1.34	<0.01
	LOC105369981	0.91	0.04	*LOC105369981*	0.89	0.04
	LOC105370404	1.16	<0.05	*LOC105370404*	1.21	0.01
	LOC105370954	0.74	<0.01	*LOC105370954*	0.79	<0.01
	LOC105372075	1.22	0.03	*LOC105372075*	1.22	0.04
	LOC105373256	0.78	0.01	*LOC105373256*	0.76	<0.01
	LOC105373496	0.81	0.03	*LOC105373496*	0.89	0.02
	LOC105374424	1.35	0.01	*LOC105374424*	1.28	0.01
	LOC105374689	1.35	0.02	*LOC105374689*	1.22	0.03
	LOC105375052	1.29	<0.01	*LOC105375052*	1.32	0.01
	LOC105375310	1.17	0.02	*LOC105375310*	1.38	<0.01
	LOC105376150	1.43	0.04	*LOC105376150*	1.42	0.04
	LOC105376203	1.14	0.02	*LOC105376203*	1.11	0.02
	LOC105376647	0.74	<0.05	*LOC105376647*	0.72	0.02
	LOC105377918	1.11	<0.01	*LOC105377918*	1.11	<0.01
	LOC105378065	0.91	0.04	*LOC105378065*	0.92	0.02
	LOC105378152	1.47	<0.01	*LOC105378152*	1.26	0.01
	LOC105378459	0.83	0.03	*LOC105378459*	0.84	0.03
	LOC105378966	1.17	0.02	*LOC105378966*	1.20	0.04
	LOC105447648	0.89	<0.05	*LOC105447648*	0.65	0.01
	LOC106699570	1.42	0.02	*LOC106699570*	1.26	0.01
	LOC730179	0.78	0.02	*LOC730179*	0.61	<0.01
	LRTM2	1.13	<0.05	*LRTM2*	1.14	0.01
	MEGF6	1.30	0.01	*MEGF6*	1.22	0.03

	MELK	0.80	<0.01	*MELK*	0.86	0.01
	MIR19B2	0.80	0.04	*MIR19B2*	0.80	<0.05
	MIR325	1.47	0.02	*MIR325*	1.47	0.03
	MIR4269	1.45	<0.01	*MIR4269*	1.75	0.02
	MIR587	1.25	0.02	*MIR587*	1.29	0.03
	MIR876	1.12	<0.05	*MIR876*	1.10	<0.05
	MIR890	1.55	<0.01	*MIR890*	1.17	0.03
	MND1	0.71	0.04	*MND1*	0.82	0.03
	MRPS28	0.67	0.01	*MRPS28*	0.77	0.02
	MUC22	0.73	0.01	*MUC22*	0.79	0.02
	MYOCD	0.80	0.03	*MYOCD*	0.86	0.04
	OACYLP	1.06	0.03	*OACYLP*	1.27	<0.01
	OR10C1	0.78	0.01	*OR10C1*	0.73	<0.01
	OR1F1	0.57	<0.01	*OR1F1*	0.70	0.01
	OR4K13	1.34	0.02	*OR4K13*	1.27	0.01
	OR51A2	0.79	0.04	*OR51A2*	0.70	0.03
	ORC1	0.63	0.01	*ORC1*	0.74	0.03
	ORM1	0.72	0.03	*ORM1*	0.77	0.04
	P2RY4	1.21	<0.05	*P2RY4*	1.27	0.02
	PCDH10	1.31	0.02	*PCDH10*	1.20	0.04
	PDE3A	0.87	<0.01	*PDE3A*	0.83	0.01
	PFKFB1	1.24	0.02	*PFKFB1*	1.21	0.04
	PLA1A	1.20	0.02	*PLA1A*	1.22	0.02
	POLR3E	0.84	<0.01	*POLR3E*	0.89	0.04
	PP2D1	1.18	0.02	*PP2D1*	1.33	0.02
	PTPMT1	0.79	0.01	*PTPMT1*	0.86	0.03
	PTPN23	0.86	0.02	*PTPN23*	0.79	0.02
	RGS7BP	1.24	0.04	*RGS7BP*	1.06	0.02
	RNASEH1	0.81	0.01	*RNASEH1*	0.89	0.03
	RPL10A	0.83	0.02	*RPL10A*	0.84	0.02
	RPL36	0.68	0.02	*RPL36*	0.75	0.02
	SF3B3	0.79	0.02	*SF3B3*	0.84	0.04
	SLA2	1.27	0.03	*SLA2*	1.16	0.02
	SLC6A5	1.24	0.04	*SLC6A5*	1.26	0.03
	SLFN14	1.20	0.01	*SLFN14*	1.43	0.03
	SMCO1	1.22	<0.01	*SMCO1*	1.28	0.02
	SP8	1.33	0.04	*SP8*	1.35	<0.05
	SPANXN3	1.24	0.01	*SPANXN3*	1.35	0.04
	SRF	0.76	0.04	*SRF*	0.82	0.04

	SRGAP3-AS3	0.85	0.02	*SRGAP3-AS3*	0.75	<0.01
	SRRD	0.79	0.02	*SRRD*	0.83	0.01
	SRXN1	0.79	0.01	*SRXN1*	0.84	0.03
	ST8SIA6-AS1	1.20	<0.01	*ST8SIA6-AS1*	1.27	<0.01
	STIMATE-MUSTN1	0.86	0.03	*STIMATE-MUSTN1*	0.85	0.01
	TARID	0.87	0.04	*TARID*	0.80	0.03
	TCERG1L-AS1	0.85	<0.05	*TCERG1L-AS1*	0.85	0.03
	THAP1	0.79	<0.01	*THAP1*	0.85	0.01
	TMEM218	0.79	<0.05	*TMEM218*	0.80	0.03
	TP53TG3HP	1.15	0.02	*TP53TG3HP*	1.28	<0.01
	TPM4	0.90	0.01	*TPM4*	0.88	0.02
	TRHDE-AS1	1.16	0.01	*TRHDE-AS1*	1.25	0.01
	TRIM64	1.17	0.03	*TRIM64*	1.22	0.02
	UBALD1	0.85	0.02	*UBALD1*	0.75	<0.01
	VWA2	1.27	<0.05	*VWA2*	1.41	0.01
	ZBTB21	1.08	0.01	*ZBTB21*	1.07	0.02
	ZC3H12A-DT	1.32	<0.01	*ZC3H12A-DT*	1.22	<0.01
	ZEB2-AS1	0.91	0.03	*ZEB2-AS1*	0.82	<0.05
	ZNF660	1.11	<0.01	*ZNF660*	1.20	0.02
	ZNRD1ASP	1.17	0.01	*ZNRD1ASP*	1.18	0.03
	ZSWIM2	1.14	0.03	*ZSWIM2*	1.19	0.03
Uncommon DEGs in JK-206 or -312 treated GC cell lines	*ACTL6B*	1.08	0.01	*AARD*	0.93	0.03
	ADAM18	0.93	0.03	*ABCC13*	1.14	0.03
	ADAM28	1.12	0.01	*ANGPT4*	1.24	<0.01
	ADGB	0.95	<0.05	*ANHX*	1.14	0.04
	ADPRS	0.86	0.03	*ANKRD26P3*	1.25	0.03
	ADRA1D	1.19	0.03	*ANKRD7*	1.16	0.04
	AGAP3	1.12	0.04	*AOAH-IT1*	0.90	0.02
	AGBL4-IT1	1.25	0.01	*ATE1-AS1*	1.32	0.01
	AGPS	0.88	<0.05	*ATG9B*	1.25	0.02
	AIRE	1.17	0.02	*AURKA*	0.78	0.03
	AK8	1.19	0.01	*AVP*	1.31	0.04
	AKR1C8	1.20	0.03	*BEND2*	1.32	<0.01
	ALDH1L1-AS2	0.87	0.01	*BEST4*	0.68	0.02
	AMMECR1	0.78	<0.01	*BHLHE22*	1.08	0.04
	ANAPC10	0.81	0.01	*BMX*	1.09	0.01
	ANKRD34C	1.23	<0.01	*BNIPL*	0.86	0.04
	APOF	1.12	0.03	*BTK*	1.17	0.02
	ARHGDIA	0.76	<0.05	*C11orf58*	0.89	0.02

	ARMC2-AS1	1.23	0.02	*C12orf73*	0.93	0.01
	ARMH4	0.81	0.03	*C1orf147*	1.22	0.02
	ASB11	0.94	0.04	*C1orf174*	0.83	0.04
	ASB5	1.16	<0.05	*C2orf92*	1.49	0.03
	ASB9	0.83	0.01	*C4orf17*	0.80	0.03
	ATP11A-AS1	0.92	0.03	*C5orf52*	1.68	0.03
	ATP13A5	0.72	0.01	*CACNA1C-IT2*	1.15	0.03
	ATP2B2	1.10	0.01	*CALHM4*	1.18	0.03
	B3GNT8	0.83	0.04	*CAPN3*	1.31	0.04
	BCL9L	1.15	0.04	*CAPN7*	0.89	0.02
	BEND3P3	1.41	0.03	*CCDC12*	0.86	<0.01
	BET1	1.29	0.04	*CCDC144NL-AS1*	0.93	0.04
	BEX1	1.31	0.04	*CCDC152*	0.88	<0.05
	BIRC5	0.63	0.04	*CCR9*	1.45	0.02
	BRINP3-DT	1.12	0.03	*CD3G*	0.87	0.04
	BTN3A2	1.17	0.04	*CD5*	1.23	0.03
	BUB1	0.82	0.01	*CDC14A*	0.87	0.03
	C1orf127	1.14	0.01	*CDH8*	1.24	0.04
	C4orf45	0.83	0.02	*CDK2AP2*	0.82	0.03
	C5orf58	1.15	0.02	*CDY1B*	0.88	0.02
	C9orf57	1.12	0.04	*CDY2B*	0.82	<0.05
	CA6	0.74	0.02	*CFAP61*	0.81	0.01
	CACNA1F	1.23	<0.05	*CHCHD10*	0.81	<0.05
	CCNA1	1.09	<0.01	*CHRNB3*	0.84	0.02
	CCND2	1.20	0.02	*CLCNKA*	0.80	<0.01
	CCT8	0.79	0.04	*CLSTN3*	1.14	0.03
	CD248	1.14	0.04	*CLUL1*	1.26	0.04
	CD300LD	1.28	0.04	*CNOT7*	0.90	0.04
	CD84	1.24	<0.05	*COL25A1-DT*	0.86	0.02
	CDH6	0.77	<0.01	*COLEC12*	1.24	0.03
	CEACAM22P	0.83	0.01	*COX4I2*	1.13	0.04
	CEACAMP10	1.16	0.01	*CRCT1*	1.39	<0.05
	CELF3	1.11	0.04	*CSMD2*	1.11	0.02
	CENPS-CORT	0.75	<0.01	*CT55*	1.05	0.03
	CFAP298	0.77	<0.01	*CT62*	1.33	0.01
	CHAC2	0.60	0.04	*CTNNA3*	1.10	0.03
	CIB3	1.11	<0.05	*CTXND1*	1.51	0.01
	CLDN14	1.31	0.02	*CWF19L1*	0.77	<0.05
	CLDN20	0.89	0.04	*CXCR2*	0.86	0.01

	CLPP	0.77	0.04	*D21S2088E*	0.80	<0.05
	CLRN1-AS1	1.17	0.02	*DAOA*	1.38	<0.05
	CLYBL-AS2	1.09	<0.05	*DBH*	1.16	0.03
	COL4A2-AS2	1.24	0.04	*DCT*	0.87	<0.05
	COMMD4	0.70	0.02	*DDC-AS1*	1.17	0.02
	CPN2	1.27	0.04	*DEFB110*	1.14	0.01
	CRNN	1.08	0.03	*DEFB135*	0.87	0.03
	CROCCP3	0.78	<0.01	*DIS3L2*	1.05	0.04
	CRX	1.17	0.01	*DKFZp451B082*	1.58	<0.05
	CSPG4P10	1.09	0.02	*DKKL1*	1.25	0.04
	CTR9	0.88	0.04	*DUSP29*	1.20	0.04
	CTXN2	1.19	<0.05	*DYNLRB2*	1.28	0.02
	CUL9	1.12	0.02	*EEF2*	0.88	0.04
	CUX1	1.14	0.01	*EHMT1*	1.09	0.04
	CXCL13	1.24	0.01	*ELOA3BP*	1.10	0.03
	CYLC2	1.24	0.01	*EPX*	1.23	<0.05
	CYP20A1	0.86	0.02	*EPYC*	0.85	<0.05
	CYP7A1	0.83	0.04	*ERVV-1*	1.19	0.02
	DANT2	1.24	0.04	*ESM1*	0.90	0.03
	DBF4B	0.87	0.01	*ESPN*	1.26	0.02
	DDX20	0.84	0.02	*F2*	1.13	0.03
	DEDD	0.89	<0.05	*FABP2*	1.32	0.03
	DENR	0.83	0.03	*FAHD2B*	1.72	0.03
	DERL3	1.23	0.03	*FAM124B*	1.17	0.03
	DHX9	0.77	0.02	*FAM167A-AS1*	1.06	0.01
	DLGAP1	1.15	0.02	*FAM186A*	1.16	0.04
	DLX2-DT	0.86	0.02	*FAM236A*	1.18	0.04
	DNAI4	1.38	0.02	*FAR2P3*	1.49	0.01
	DNAJB13	1.27	0.04	*FBXL21P*	1.16	0.04
	DPP9	0.83	0.04	*FBXO47*	0.85	0.01
	DRC1	1.14	0.03	*FCAMR*	0.77	<0.05
	DSCAM-IT1	0.81	0.03	*FCGR3B*	1.44	<0.05
	DSCR10	1.25	0.01	*FCN1*	1.15	0.01
	EFCAB9	0.88	0.02	*FGL2*	1.23	<0.05
	EIF1AX	0.74	<0.05	*FKBP9P1*	0.76	<0.01
	EIF4E1B	1.12	0.01	*FLJ41941*	0.72	0.02
	ELAVL1	0.77	0.03	*FOLH1B*	1.09	0.01
	ELMO2P1	1.36	0.02	*FOXR1*	1.22	0.03
	ELOVL2-AS1	0.76	0.01	*FREM2-AS1*	1.33	<0.01

	EMC8	0.78	0.01	*FXYD1*	1.21	0.03
	ENPP3	1.31	<0.05	*GARIN4*	1.18	0.02
	EOLA1-DT	1.31	0.02	*GLRB*	0.85	0.03
	ERMN	1.25	0.04	*GOLGA6L7*	1.70	0.01
	ESPL1	0.81	0.02	*GP9*	1.25	0.02
	FABP5P3	0.79	0.02	*GPR12*	0.95	<0.01
	FAM172BP	1.12	0.02	*GPR182*	1.13	0.04
	FAM90A20P	1.13	0.03	*GREM2*	1.17	0.03
	FBXL22	1.13	<0.01	*GRIFIN*	1.19	0.02
	FBXO5	0.66	0.01	*GRIK1*	0.83	0.02
	FCN2	0.61	0.03	*GRIN2C*	1.10	<0.05
	FGD3	1.37	0.04	*GRM3*	1.03	0.01
	FGD5P1	0.81	0.01	*GRM8*	0.85	0.04
	FLJ13224	0.86	0.02	*GSTT4*	0.72	<0.01
	FLJ16171	1.20	0.02	*GTSE1-DT*	1.12	<0.01
	FLJ31183	1.19	0.03	*GUCA1C*	0.90	0.03
	FOXD4L5	1.24	<0.01	*GUCA2B*	1.13	<0.05
	FRG1	0.88	0.02	*H4C4*	0.66	0.02
	FRRS1L	0.88	0.04	*HBM*	1.13	0.02
	FUT7	1.29	0.01	*HCG26*	1.18	<0.01
	GABRQ	0.78	0.04	*HCG9*	1.14	0.02
	GAS2L1P2	1.28	<0.01	*HEPN1*	1.18	<0.01
	GASK1A	1.21	0.03	*HEXD-IT1*	0.79	0.04
	GDF7	1.23	0.02	*HINT1*	0.76	0.01
	GMIP	1.18	0.04	*HLA-DQB2*	1.09	<0.01
	GOLGA8IP	1.20	0.04	*HLA-F-AS1*	0.75	0.03
	GPA33	1.21	0.03	*HTR1F*	1.31	<0.05
	GPATCH4	0.69	0.03	*IDO1*	0.81	0.04
	GPR158-AS1	0.78	<0.01	*IFNA7*	1.42	0.02
	GSTA3	1.36	0.02	*IFNA8*	1.24	0.02
	GTF2F1	0.85	0.03	*IGHV1OR15-9*	1.33	0.04
	GTF2IRD2B	1.18	0.01	*IGHV3-35*	1.31	0.03
	H1-2	0.68	0.03	*IGLJ2*	1.18	<0.05
	H2AC1	1.42	0.04	*IGLV7-43*	0.67	0.02
	H2AC13	0.53	0.02	*IMPG1*	0.84	<0.01
	H2AC16	0.74	<0.05	*INPP5J*	0.80	0.03
	H2AC17	0.52	0.02	*ISLR*	1.19	<0.01
	H2AC4	0.77	<0.05	*ITIH1*	0.70	0.02
	H2BC10	0.61	0.04	*ITIH2*	0.78	0.02

	HIST1H2BK	0.65	0.04	*KCNK9*	1.31	0.04
	H2BC13	0.60	0.01	*KCNMB2*	0.80	0.01
	H3C11	0.59	0.02	*KLF16*	0.83	<0.05
	H3C15	0.61	<0.05	*KLHL31*	1.41	0.02
	H3C4	0.78	<0.05	*KRT32*	1.06	0.02
	H3C7	0.63	0.03	*KRT40*	0.85	0.03
	H4C12	0.73	0.01	*KRT79*	1.28	0.01
	HASPIN	0.77	0.04	*KRT9*	1.32	0.02
	HEPACAM2	0.88	0.03	*KRTAP11-1*	1.12	0.04
	HMCN2	1.10	0.02	*KRTAP15-1*	0.81	<0.05
	HORMAD2-AS1	0.78	<0.05	*KRTAP19-7*	1.21	0.04
	HSD11B1	1.06	0.02	*KRTAP21-2*	0.84	0.01
	HSP90B3P	0.73	0.01	*KRTAP4-11*	0.63	0.02
	HSPBP1	0.74	0.01	*L3MBTL2*	0.84	0.03
	HTR5A-AS1	0.85	<0.05	*LAGE3*	0.83	<0.01
	IGF1	0.91	0.04	*LAMA5-AS1*	1.20	0.02
	IGFALS	1.11	0.04	*LANCL1-AS1*	0.77	0.01
	IL36A	0.76	0.03	*LARP7*	0.90	<0.05
	INTS4P2	0.82	0.02	*LCE1A*	0.68	0.02
	IRAG1-AS1	1.25	0.01	*LCE1B*	1.25	<0.01
	KATNBL1P6	0.76	0.02	*LCE3A*	1.20	0.04
	KCNH6	1.31	0.04	*LCE3C*	0.74	0.01
	KCTD2	0.90	0.04	*LCN9*	0.72	0.02
	KIF15	0.77	0.02	*LCTL*	0.82	0.04
	KIF18A	0.79	0.04	*LEMD1-AS1*	0.75	0.01
	KIR2DS2	0.62	0.01	*LGALS2*	1.22	0.04
	KIR2DS3	0.86	0.01	*LHFPL7*	1.23	<0.01
	KLF17	0.81	0.02	*LILRA3*	1.14	0.01
	KLHDC8A	1.30	0.04	*LIN7A*	1.23	0.04
	KNG1	1.29	0.03	*LINC00307*	0.82	0.01
	KRTAP24-1	0.91	0.04	*LINC00400*	0.91	0.04
	KRTAP29-1	0.81	0.01	*LINC00485*	0.78	0.01
	KRTAP4-3	1.39	0.01	*LINC00544*	0.84	0.03
	KRTAP4-5	0.83	<0.05	*LINC00566*	1.14	0.04
	LAG3	0.88	0.04	*LINC00570*	0.87	0.03
	LBP	0.79	0.03	*LINC00595*	0.80	0.03
	LCE3B	1.19	0.01	*LINC00706*	0.54	0.03
	LDB2	1.27	0.03	*LINC00927*	1.09	0.02
	LEO1	0.85	0.02	*LINC00933*	1.32	0.01

	LINC00276	1.25	0.01	*LINC01081*	0.87	0.04
	LINC00304	1.46	0.02	*LINC01114*	1.31	0.01
	LINC00385	0.91	0.02	*LINC01151*	1.25	<0.05
	LINC00398	1.29	0.01	*LINC01251*	1.28	0.02
	LINC00502	1.08	0.04	*LINC01276*	0.89	0.04
	LINC00523	1.15	0.03	*LINC01364*	1.28	0.03
	LINC00609	0.93	0.03	*LINC01423*	1.21	0.03
	LINC00905	0.84	0.02	*LINC01451*	0.94	0.02
	LINC00974	1.25	0.03	*LINC01467*	0.83	0.04
	LINC01078	0.88	0.04	*LINC01475*	1.27	<0.05
	LINC01088	1.20	0.03	*LINC01496*	0.86	0.03
	LINC01115	0.91	0.04	*LINC01546*	1.67	<0.05
	LINC01237	1.12	0.01	*LINC01566*	1.35	0.01
	LINC01335	1.12	0.02	*LINC01579*	1.12	0.03
	LINC01340	1.15	0.01	*LINC01618*	0.87	0.02
	LINC01493	0.86	0.04	*LINC01869*	0.94	<0.05
	LINC01517	0.87	0.03	*LINC02209*	1.26	<0.01
	LINC01816	0.83	0.03	*LINC02587*	0.84	0.04
	LINC02872	1.22	0.02	*LINC02694*	1.23	<0.01
	LINC02907	1.15	0.03	*LOC100130458*	1.19	<0.05
	LIPI	0.84	0.04	*LOC100505622*	0.87	0.01
	LNPEP	1.18	0.03	*LOC100505685*	1.13	0.03
	LOC100101148	0.80	0.04	*LOC100506725*	1.19	0.04
	LOC100128437	1.34	0.01	*LOC100506985*	1.13	<0.01
	LOC100128573	1.23	0.04	*LOC100996637*	0.73	0.03
	LOC100129434	1.27	0.04	*LOC101060385*	0.88	0.02
	LOC100129596	0.86	0.02	*LOC101927059*	1.27	<0.05
	LOC100130507	0.80	0.03	*LOC101927082*	1.12	0.03
	LOC100133106	0.84	0.04	*LOC101927120*	1.25	0.01
	LOC100288570	1.06	0.04	*LOC101927153*	0.80	0.04
	LOC100506489	1.27	0.01	*LOC101927365*	0.82	<0.05
	LOC100506928	1.09	0.03	*LOC101927412*	1.33	0.02
	LOC100507661	0.84	<0.05	*LOC101927434*	0.79	0.04
	LOC100652871	1.24	<0.01	*LOC101927435*	0.87	0.02
	LOC100653233	1.21	0.04	*LOC101927468*	0.60	0.01
	LOC100996419	1.34	0.02	*LOC101927505*	1.26	0.02
	LOC100996630	1.27	0.04	*LOC101927664*	0.85	0.01
	LOC101060391	0.80	0.01	*LOC101927721*	1.22	0.04
	LOC101926948	1.12	0.04	*LOC101927766*	0.77	0.02

	LOC101927023	1.44	<0.01	*LOC101927853*	1.30	0.03
	LOC101927346	0.72	0.02	*LOC101928058*	1.16	0.03
	LOC101927378	1.15	0.02	*LOC101928077*	0.93	<0.05
	LOC101927467	0.83	0.02	*LOC101928161*	1.14	<0.05
	LOC101927502	1.31	<0.05	*LOC101928174*	1.26	0.03
	LOC101927635	0.85	0.01	*LOC101928177*	0.70	0.02
	LOC101927694	1.16	0.02	*LOC101928253*	1.24	0.04
	LOC101927768	0.93	<0.05	*LOC101928306*	0.67	0.03
	LOC101927769	1.22	<0.05	*LOC101928404*	0.72	<0.05
	LOC101927798	0.80	0.01	*LOC101928409*	0.88	0.02
	LOC101927851	1.22	0.04	*LOC101928418*	1.38	0.04
	LOC101927972	0.79	0.04	*LOC101928561*	1.24	0.02
	LOC101928092	1.47	0.04	*LOC101928595*	1.28	0.03
	LOC101928111	1.40	0.01	*LOC101929058*	0.77	0.03
	LOC101928188	1.70	0.04	*LOC101929084*	1.11	0.03
	LOC101928439	0.85	<0.05	*LOC101929485*	1.14	<0.05
	LOC101928472	0.76	0.01	*LOC101929526*	1.52	0.01
	LOC101928711	0.93	0.02	*LOC101929538*	1.20	<0.05
	LOC101928721	1.20	<0.05	*LOC101929563*	1.12	0.01
	LOC101928841	1.15	0.04	*LOC101930114*	1.14	<0.05
	LOC101928877	1.14	0.04	*LOC102467216*	0.80	0.01
	LOC101928896	0.90	0.03	*LOC102477328*	0.78	0.03
	LOC101929140	1.62	0.03	*LOC102723418*	0.87	0.04
	LOC101929153	1.24	<0.01	*LOC102723640*	1.14	<0.05
	LOC101929432	1.09	0.03	*LOC102723757*	0.90	0.03
	LOC101929445	1.33	0.03	*LOC102724247*	0.83	0.04
	LOC101929657	1.18	0.02	*LOC102724380*	0.68	0.01
	LOC101929697	0.87	0.04	*LOC102724443*	0.89	<0.01
	LOC101929723	1.47	0.03	*LOC102724465*	0.87	0.02
	LOC102723313	1.25	0.01	*LOC105369391*	1.17	0.01
	LOC102723714	1.42	0.04	*LOC105369406*	0.83	0.01
	LOC102724509	0.81	0.01	*LOC105369413*	1.12	<0.01
	LOC102724670	0.82	0.01	*LOC105369527*	1.29	0.03
	LOC102724698	1.43	0.03	*LOC105369541*	1.19	0.02
	LOC102724861	1.21	0.01	*LOC105369639*	1.22	0.02
	LOC102725072	1.58	0.04	*LOC105369893*	0.79	0.02
	LOC105369361	0.82	0.01	*LOC105370039*	0.90	0.03
	LOC105369539	1.30	<0.05	*LOC105370120*	1.12	0.04
	LOC105369549	0.94	0.03	*LOC105370149*	0.82	0.04

	LOC105369859	0.94	0.04	*LOC105370186*	0.80	0.02
	LOC105369926	0.81	0.04	*LOC105370232*	1.22	0.02
	LOC105369971	0.93	0.04	*LOC105370344*	1.14	<0.05
	LOC105369984	0.82	0.03	*LOC105370456*	0.83	0.01
	LOC105370058	0.78	<0.01	*LOC105370492*	1.23	<0.01
	LOC105370088	1.24	0.04	*LOC105370539*	0.80	0.01
	LOC105370129	1.19	0.04	*LOC105370560*	0.79	<0.05
	LOC105370230	1.34	0.02	*LOC105370616*	1.27	<0.01
	LOC105370319	0.82	0.03	*LOC105370767*	1.26	0.03
	LOC105370370	0.91	0.03	*LOC105371195*	1.26	0.02
	LOC105370415	1.18	<0.01	*LOC105371301*	0.74	<0.05
	LOC105370632	0.78	0.03	*LOC105371357*	1.27	0.01
	LOC105370745	0.74	0.02	*LOC105371541*	0.90	0.04
	LOC105370781	1.10	<0.01	*LOC105371560*	1.11	<0.05
	LOC105370962	1.18	<0.05	*LOC105371600*	1.29	0.03
	LOC105371317	0.84	0.03	*LOC105371750*	1.15	0.03
	LOC105371379	1.10	<0.01	*LOC105371760*	1.22	0.01
	LOC105371528	1.21	0.02	*LOC105371976*	1.16	0.01
	LOC105371784	0.83	0.02	*LOC105371979*	1.17	<0.05
	LOC105371824	1.13	0.04	*LOC105372132*	1.48	0.02
	LOC105371974	1.42	0.02	*LOC105372155*	1.17	0.03
	LOC105372009	1.16	<0.05	*LOC105372258*	1.17	0.01
	LOC105372202	1.14	<0.01	*LOC105372330*	0.75	<0.01
	LOC105372430	1.25	0.02	*LOC105372569*	1.15	0.04
	LOC105372587	1.38	0.04	*LOC105372596*	0.83	<0.01
	LOC105372598	1.31	<0.05	*LOC105372929*	0.69	0.03
	LOC105372649	1.19	0.04	*LOC105373180*	1.15	0.03
	LOC105372676	0.85	0.01	*LOC105373218*	0.85	0.04
	LOC105372763	1.12	<0.01	*LOC105373249*	1.13	0.02
	LOC105372876	0.73	0.01	*LOC105373400*	0.87	0.02
	LOC105372999	1.06	0.02	*LOC105373508*	1.21	0.04
	LOC105373011	1.44	0.02	*LOC105373522*	1.26	<0.01
	LOC105373082	1.19	0.01	*LOC105373530*	0.77	0.03
	LOC105373175	0.88	0.02	*LOC105373585*	1.04	<0.05
	LOC105373484	0.85	<0.05	*LOC105373615*	1.22	0.04
	LOC105373502	1.09	0.04	*LOC105373617*	0.84	0.01
	LOC105373586	1.29	0.01	*LOC105373784*	0.91	0.03
	LOC105373597	1.12	0.04	*LOC105373789*	0.84	0.03
	LOC105373772	0.70	0.01	*LOC105373899*	1.12	0.03

	LOC105374005	1.14	0.02	*LOC105374229*	0.69	0.03
	LOC105374024	0.80	0.03	*LOC105374265*	0.78	0.03
	LOC105374201	1.17	<0.05	*LOC105374546*	1.17	0.02
	LOC105374325	0.75	0.01	*LOC105374617*	0.89	0.03
	LOC105374623	0.90	0.02	*LOC105374643*	1.19	<0.05
	LOC105374763	0.87	<0.05	*LOC105374644*	1.10	<0.05
	LOC105374793	0.79	<0.01	*LOC105374645*	1.23	0.02
	LOC105375287	0.80	0.01	*LOC105374728*	1.25	0.01
	LOC105375341	0.78	0.02	*LOC105374729*	1.14	0.01
	LOC105375429	1.31	0.03	*LOC105374753*	1.11	<0.01
	LOC105375523	1.21	0.04	*LOC105374843*	1.22	0.04
	LOC105375635	0.82	<0.05	*LOC105374905*	0.81	0.03
	LOC105375666	1.45	0.01	*LOC105375132*	0.86	0.04
	LOC105375847	1.23	0.02	*LOC105375152*	0.77	0.01
	LOC105375937	1.15	0.01	*LOC105375224*	0.70	0.04
	LOC105376130	0.88	0.02	*LOC105375547*	0.79	0.03
	LOC105376412	0.85	0.01	*LOC105375589*	1.11	0.03
	LOC105376605	0.78	0.04	*LOC105375710*	1.11	0.04
	LOC105376653	1.13	<0.05	*LOC105375861*	1.20	0.01
	LOC105376706	1.17	0.01	*LOC105376020*	0.86	<0.05
	LOC105376789	0.87	0.03	*LOC105376043*	1.17	0.04
	LOC105376975	0.78	0.02	*LOC105376063*	1.15	0.03
	LOC105377026	1.08	0.02	*LOC105376101*	0.85	0.04
	LOC105377061	0.73	0.03	*LOC105376685*	1.22	0.01
	LOC105377105	0.76	0.02	*LOC105376928*	1.37	0.04
	LOC105377295	1.32	<0.05	*LOC105377003*	1.22	<0.01
	LOC105377473	1.14	0.01	*LOC105377041*	0.67	0.01
	LOC105378047	1.21	<0.01	*LOC105377115*	0.91	0.02
	LOC105378280	1.11	0.01	*LOC105377127*	1.14	0.04
	LOC105378516	1.24	0.02	*LOC105377145*	0.89	<0.01
	LOC105378641	1.15	0.04	*LOC105377153*	0.81	0.03
	LOC105378654	0.92	0.04	*LOC105377303*	1.17	0.04
	LOC105378702	1.34	0.01	*LOC105377505*	0.83	0.01
	LOC105378706	1.23	0.03	*LOC105377742*	0.84	<0.05
	LOC105378738	0.80	0.04	*LOC105377755*	0.83	0.04
	LOC105378769	1.23	<0.05	*LOC105377774*	1.19	0.04
	LOC105378839	0.77	0.04	*LOC105378309*	0.89	<0.05
	LOC105379091	1.27	0.03	*LOC105378334*	1.11	<0.01
	LOC105379592	1.28	0.02	*LOC105378564*	1.12	0.01

	LOC105379881	1.32	<0.01	*LOC105378612*	0.92	0.04
	LOC153910	0.67	0.01	*LOC105378618*	1.12	0.04
	LOC255654	1.25	0.02	*LOC105378727*	1.11	<0.01
	LOC339975	1.19	0.03	*LOC105378784*	0.91	0.04
	LOC400620	1.08	0.03	*LOC105378997*	1.10	0.01
	LOC400800	1.26	0.02	*LOC105379175*	0.74	0.04
	LOC643802	1.31	0.01	*LOC105379379*	1.25	0.02
	LOC729461	0.73	<0.05	*LOC105755953*	0.76	0.03
	LRIT3	0.80	0.04	*LOC149950*	1.17	0.03
	LRP4-AS1	0.80	<0.05	*LOC339902*	0.82	<0.05
	LRRC15	1.32	0.03	*LOC401176*	0.73	0.03
	LRRC30	1.20	0.02	*LOC440602*	0.84	0.04
	LRRC40	0.91	0.04	*LOC494141*	0.89	0.03
	LUC7L3	1.26	0.02	*LOC642574*	1.14	0.04
	MAP3K15	0.91	0.01	*LOC643441*	1.09	0.02
	MAP4K1	0.82	0.04	*LOC650293*	0.75	0.04
	MCM10	0.69	0.03	*LRRC32*	1.46	0.04
	MEG3	0.82	0.02	*LUNAR1*	0.77	0.04
	MEP1A	0.67	0.01	*LYZL6*	0.79	0.02
	MIB1	1.15	<0.05	*MAGEB6*	0.72	0.01
	MIR1237	0.64	0.04	*MAPT-AS1*	1.28	<0.05
	MIR1269A	0.83	0.04	*MCPH1-AS1*	1.17	0.01
	MIR2115	1.23	<0.01	*ME3*	1.10	<0.01
	MIR217HG	1.15	0.02	*MIR103B1*	0.90	0.03
	MIR26B	1.28	0.04	*MIR103B2*	0.70	0.03
	MIR3119-2	0.86	0.04	*MIR105-1*	0.89	0.02
	MIR3120	0.77	0.01	*MIR106A*	0.89	<0.05
	MIR3129	0.82	0.03	*MIR1283-1*	0.75	0.02
	MIR3173	1.11	0.02	*MIR146A*	1.10	0.04
	MIR324	0.88	<0.01	*MIR2052HG*	0.84	0.01
	MIR3671	1.89	0.04	*MIR2053*	0.82	0.02
	MIR3915	0.81	0.04	*MIR3150A*	1.16	0.04
	MIR3922	1.23	0.04	*MIR3156-1*	0.81	0.01
	MIR4266	0.47	0.03	*MIR3158-1*	0.77	<0.05
	MIR4276	0.73	0.03	*MIR3198-1*	0.80	0.01
	MIR4294	1.25	0.01	*MIR320C2*	1.51	0.02
	MIR4487	0.78	0.04	*MIR342*	1.13	0.03
	MIR4645	1.36	0.02	*MIR3659*	0.91	0.04
	MIR4797	1.26	0.04	*MIR3910-1*	0.91	0.03

	MIR499A	1.32	0.03	*MIR4279*	0.54	0.03
	MIR507	1.28	0.03	*MIR4301*	1.23	0.04
	MIR516B2	0.53	0.04	*MIR432*	1.34	0.01
	MIR519C	0.95	0.02	*MIR4476*	1.14	0.04
	MIR548W	0.83	<0.05	*MIR4477A*	1.15	<0.05
	MIR550B2	1.43	0.04	*MIR4483*	1.20	0.03
	MIR551B	1.17	0.04	*MIR4499*	0.64	<0.05
	MIR670	1.39	0.03	*MIR4662A*	0.86	0.02
	MIR767	0.84	0.01	*MIR497HG*	0.75	0.03
	MIR92B	0.78	<0.05	*MIR498*	1.15	0.04
	MIR934	0.86	0.01	*MIR513B*	0.77	0.03
	MIRLET7A2	1.35	0.04	*MIR516B1*	0.75	0.02
	MPC1L	0.83	0.04	*MIR520A*	1.48	<0.05
	MPLKIP	1.20	<0.05	*MIR527*	1.60	0.03
	MRLN	1.19	0.01	*MIR548T*	2.11	0.02
	MRPL12	0.75	0.02	*MIR7-3*	1.10	0.02
	MRPS7	0.78	0.03	*MIXL1*	1.20	<0.01
	MS4A12	1.19	0.03	*MLH1*	0.83	0.04
	MSH6	0.94	0.04	*MLIP-IT1*	1.18	0.04
	MST1	1.40	0.01	*MPP7*	1.23	0.04
	MTBP	0.84	0.01	*MRGPRX2*	0.78	0.04
	MTRNR2L6	1.25	0.03	*MRM1*	0.80	0.02
	NAPSB	0.80	0.02	*MROH9*	1.20	0.04
	NCL	0.82	0.02	*MRPL19*	0.87	0.04
	NEGR1-IT1	0.85	0.02	*MS4A15*	1.19	0.03
	NGRN	0.84	0.04	*MTA2*	0.86	0.04
	NID2	1.22	<0.05	*MTUS2-AS1*	1.21	0.04
	NKX6-1	1.25	<0.05	*MUC19*	1.14	0.03
	NNT-AS1	1.16	0.04	*MUC6*	1.10	0.04
	NOL11	0.74	<0.05	*NCF1C*	0.77	0.01
	NPAP1	1.25	0.03	*NEIL2*	0.90	0.04
	NPB	0.76	0.01	*NEXN-AS1*	1.16	<0.05
	NR2F2	0.73	0.01	*NFAM1*	0.87	0.02
	NRIR	1.19	0.03	*NME9*	1.15	0.03
	NUDT21	0.81	<0.05	*NNAT*	1.13	0.03
	NUDT8	0.90	0.04	*NOB1*	0.81	0.03
	NUS1	0.85	0.04	*NPR3*	1.15	0.02
	NUTM1	0.79	<0.05	*NTRK3*	1.19	<0.05
	NXF4	0.76	0.03	*NUTM2D*	1.24	0.04

	OAZ3	0.82	<0.01	*OR10G2*	0.84	<0.05
	OLIG1	0.92	0.02	*OR14I1*	0.89	0.04
	OLIG2	0.70	0.03	*OR2F1*	1.14	0.03
	OOSP2	0.88	<0.05	*OR2H2*	1.32	0.02
	OPN1MW	1.17	0.02	*OR2T11*	0.81	0.03
	OR10H4	0.75	0.03	*OR2T8*	1.06	<0.05
	OR10W1	1.15	0.01	*OR52B4*	0.86	0.04
	OR10Z1	0.77	0.01	*OR56A1*	0.75	0.04
	OR2S2	1.38	<0.05	*OR6C2*	1.21	0.01
	OR2V2	0.77	<0.05	*OR6F1*	0.84	0.02
	OR3A2	0.83	0.03	*OR6M1*	0.82	0.03
	OR4D10	1.25	0.02	*OVOL3*	0.82	<0.01
	OR4D9	0.83	0.01	*PAX2*	0.86	0.01
	OR4F15	0.78	0.01	*PDHX*	0.85	0.04
	OR4L1	0.73	<0.01	*PDILT*	1.26	0.03
	OR6C1	1.20	0.02	*PDZD7*	1.22	0.01
	OR7A5	0.82	0.02	*PEX26*	0.84	0.03
	ORMDL3	1.28	0.01	*PGBP*	1.23	0.01
	PABPC1L2B-AS1	0.94	0.02	*PGM2*	0.89	<0.05
	PACSIN3	0.89	<0.01	*PHF10*	0.87	0.02
	PARP1	0.74	0.04	*PIGR*	0.85	0.02
	PCNA-AS1	0.68	<0.05	*PKN2-AS1*	0.92	0.03
	PDE1A	0.87	0.02	*PNLIPRP3*	1.14	0.04
	PDX1	0.89	0.02	*PPDPFL*	0.83	0.04
	PGLYRP4	0.89	0.04	*PPP1R2B*	0.71	0.03
	PHF5A	0.78	<0.01	*PRKX-AS1*	0.75	0.01
	PIEZO2	1.07	<0.01	*PRRX2-AS1*	1.20	0.01
	PLPBP	0.79	0.01	*PTCH2*	1.08	0.01
	PLSCR5	0.84	0.02	*PTPRD-AS1*	0.85	0.01
	PNPLA5	0.76	0.03	*PYY*	0.82	0.02
	POLA2	0.85	0.03	*RAB41*	0.85	<0.05
	POLD3	0.83	0.03	*RADIL*	1.37	0.03
	POLDIP3	0.78	0.01	*RAP2A*	0.83	<0.05
	POM121L12	1.21	0.03	*RBFADN*	1.17	0.04
	PPAT	0.77	0.04	*RBM46*	0.90	0.03
	PPP2R5D	0.71	0.02	*REXO1*	0.88	0.04
	PRMT3	0.81	0.01	*RFTN1*	0.90	<0.05
	PRX	1.13	<0.01	*RFX1*	0.93	0.03
	PSG8	0.83	0.02	*RHBG*	1.17	<0.05

	PTGES3	0.85	0.04	*RIMBP3B*	0.92	<0.05
	PTGIR	1.09	0.04	*RIPPLY3*	0.86	0.04
	RAB9BP1	0.70	0.01	*RLN1*	1.37	0.03
	RBBP8	0.85	0.02	*RMDN2*	0.87	0.03
	RBM44	1.19	<0.01	*RMDN2-AS1*	0.76	0.03
	RBM48	1.09	0.02	*RNF208*	0.79	0.04
	RGL4	0.86	0.01	*RNF214*	0.89	0.04
	RHOA	0.92	<0.05	*RPL21*	0.83	<0.05
	RPL34-DT	1.24	0.01	*RPL32*	0.74	0.01
	RPS19BP1	0.82	0.03	*RPS16*	0.85	0.03
	RPS6KA2-AS1	1.12	<0.01	*RPS18*	0.85	<0.01
	RPSAP52	0.75	0.03	*RRP9*	0.81	0.02
	RPUSD2	0.76	0.03	*SCARF2*	1.16	0.03
	RRP12	0.85	0.04	*SCG5*	1.14	0.04
	RRP8	0.75	0.02	*SDCBPP2*	1.22	0.01
	RSPO1	1.21	0.01	*SELENOH*	0.85	0.04
	RUVBL2	0.72	0.02	*SF3A2*	0.83	0.04
	RXRG	0.88	0.04	*SIGLEC11*	1.39	<0.01
	SAAL1	0.79	<0.05	*SIGLECL1*	0.84	0.01
	SAP18	0.76	<0.01	*SIT1*	1.22	<0.01
	SCOC-AS1	1.21	0.01	*SLC22A12*	0.92	0.01
	SCTR	1.18	0.01	*SLC27A3*	0.90	0.04
	SDC4	1.34	0.04	*SLC35G3*	1.24	0.02
	SEC14L2	1.28	0.03	*SLC35G5*	1.32	0.03
	SEC24C	0.95	0.04	*SLC38A3*	0.88	0.03
	SEC31B	1.37	0.02	*SLC49A3*	0.88	0.01
	SEPTIN14P20	1.48	0.02	*SLC7A3*	0.86	0.03
	SERPINA4	0.89	0.04	*SMAD1-AS2*	0.67	<0.01
	SET	0.69	0.01	*SMTNL2*	1.21	0.02
	SETD4	1.17	<0.05	*SNORA30*	1.42	0.04
	SFMBT2	1.32	0.01	*SNORD113-7*	0.81	0.02
	SFTPB	1.30	0.03	*SNORD114-6*	1.77	<0.05
	SGF29	0.71	0.01	*SNORD116-30*	1.45	<0.05
	SIGLEC6	0.84	0.02	*SNORD116-5*	1.25	0.03
	SIRPD	1.18	0.04	*SNRPEP2*	0.82	0.02
	SKA3	0.64	0.04	*SPATA32*	1.30	0.04
	SLA	1.11	<0.05	*SPDYE3*	0.50	0.02
	SLAMF1	1.15	0.04	*SPG21*	0.82	<0.05
	SLC27A1	1.36	0.01	*SPO11*	1.10	0.01

	SLC5A8	1.25	<0.05	*SPRNP1*	0.86	0.01
	SMARCB1	0.82	0.03	*SPRR2C*	1.25	<0.01
	SMDT1	0.86	0.04	*SPRR4*	1.19	0.04
	SMIM18	1.15	<0.05	*STMN2*	0.70	0.02
	SNORA35	1.15	<0.05	*STMN4*	1.40	0.01
	SNORD59B	1.38	0.03	*SUMO1*	1.36	0.03
	SNRNP40	0.81	0.03	*TAC4*	1.18	0.04
	SNRPD2	0.78	0.03	*TAF1A*	1.19	0.01
	SNRPD3	0.76	<0.05	*TAT-AS1*	1.24	0.02
	SNRPF	0.68	0.04	*TBX15*	1.20	0.02
	SPANXN4	1.41	0.01	*TDGF1P3*	1.33	0.01
	SPATA31A1	1.06	<0.05	*TEX29*	1.42	0.02
	SPON1	1.28	0.01	*TLR7*	1.17	<0.05
	SRGAP2-AS1	0.69	<0.01	*TM4SF18*	1.19	0.01
	SS18L2	0.84	0.04	*TMEM141*	0.89	0.02
	ST7-AS2	0.83	0.01	*TMEM212-AS1*	1.17	0.01
	STEAP1B	1.21	0.01	*TMEM244*	1.23	0.04
	SUCLA2-AS1	0.71	0.04	*TMEM26-AS1*	0.91	0.04
	SYNJ2	1.11	0.02	*TMEM30CP*	1.13	0.01
	TAF3	0.79	0.03	*TMEM72-AS1*	0.75	0.02
	TAS2R16	1.33	0.03	*TMEM88B*	0.87	0.02
	TASL	1.22	0.01	*TNFSF11*	1.13	0.04
	TBL2	0.79	0.04	*TNK2-AS1*	0.94	0.01
	TEX22	0.83	0.01	*TPI1P3*	1.49	0.04
	TEX28	0.91	<0.05	*TRAV12-3*	1.28	0.01
	TEX35	1.12	<0.05	*TRAV7*	0.86	0.02
	TEX41	0.84	0.02	*TRAV8-7*	0.90	0.04
	TGM1	0.83	<0.01	*TRBV10-2*	0.77	0.02
	THEMIS	0.85	<0.05	*TRBV7-4*	1.41	0.01
	THOP1	0.85	<0.01	*TTLL1*	1.26	0.03
	TIFAB	1.34	0.01	*UTF1*	1.18	0.02
	TK2	1.22	0.01	*VENTX*	1.19	<0.01
	TM6SF1	1.27	0.04	*VSTM2A-OT1*	1.18	0.01
	TMEM132E	1.17	0.03	*WDR88*	0.87	0.04
	TMEM140	1.47	0.03	*WHAMMP3*	1.15	0.01
	TMEM252	0.78	0.03	*WNT7A*	0.70	0.03
	TMEM40	1.21	<0.05	*XLOC_009911*	1.52	0.01
	TMEM41B	1.31	0.03	*ZNF157*	1.61	0.02
	TMEM70	0.86	0.03	*ZNF322*	0.92	<0.01

	TMEM74	0.79	0.04	*ZNF667*	0.90	0.03
	TNFRSF17	1.12	0.04	*ZPLD1*	0.87	<0.05
	TOMM40	0.60	0.04			
	TRAJ13	1.31	<0.05			
	TRAJ37	1.41	0.02			
	TRAJ56	1.26	<0.05			
	TRAJ9	1.55	0.04			
	TRDJ2	1.22	0.02			
	TRIM49B	0.85	0.03			
	TRIM51EP	1.16	<0.05			
	TRIM68	1.13	0.03			
	TRMO	0.93	0.03			
	TRMT44	1.11	<0.05			
	TRPM3	0.94	0.04			
	TSPO2	1.26	0.02			
	TSR3	0.77	0.03			
	TTC3	1.21	0.03			
	TTTY5	0.82	0.03			
	TUBA3D	0.81	<0.01			
	TUBA4B	0.82	0.01			
	TUSC1	0.83	0.04			
	UBE2D2	0.78	0.01			
	UBE2I	0.77	<0.05			
	UBE2S	0.66	0.02			
	UBTFL1	0.73	0.03			
	UGT2A2	1.35	0.02			
	UNC13C	1.22	0.04			
	USH1G	0.80	<0.05			
	USP1	0.78	0.01			
	USP17L10	1.34	<0.01			
	VSX2	0.91	0.01			
	VWA5B1	0.72	0.02			
	WFDC1	1.16	0.03			
	WFDC13	1.32	0.02			
	WFIKKN2	1.11	0.04			
	YDJC	0.81	0.04			
	ZBTB9	0.77	0.02			
	ZC3H18-AS1	1.15	<0.01			
	ZCCHC10	0.81	<0.01			

	ZKSCAN1	1.25	0.04			
	ZNF273	1.20	0.03			
	ZNF365	1.23	0.04			
	ZNF436-AS1	1.17	<0.01			
	ZNF549	1.34	0.02			
	ZNF579	0.95	0.03			
	ZNF593	0.77	0.01			
	ZNF677	0.74	0.02			
	ZNF705A	1.31	0.04			
	ZNF705D	0.80	0.04			
	ZNF771	0.87	0.04			
	ZNF781	1.18	<0.05			
	ZNF833P	1.45	0.03			
	ZNF876P	1.45	0.01			
	ZNF91	1.19	0.03			

Supplemental References

1. Kim, J.H.; Park, S.; Lim, S.M.; Eom, H.J.; Balch, C.; Lee, J.; Kim, G.J.; Jeong, J.H.; Nam, S.; Kim, Y.H. Rational design of small molecule RHOA inhibitors for gastric cancer. *Pharmacogenomics J* **2020**, *20*, 601-612, doi:10.1038/s41397-020-0153-6.
2. Barretina, J.; Caponigro, G.; Stransky, N.; Venkatesan, K.; Margolin, A.A.; Kim, S.; Wilson, C.J.; Lehar, J.; Kryukov, G.V.; Sonkin, D.; et al. The Cancer Cell Line Encyclopedia enables predictive modelling of anticancer drug sensitivity. *Nature* **2012**, *483*, 603-607, doi:10.1038/nature11003.
3. Ikari, N.; Serizawa, A.; Tanji, E.; Yamamoto, M.; Furukawa, T. Analysis of RHOA mutations and their significance in the proliferation and transcriptome of digestive tract cancer cells. *Oncol Lett* **2021**, *22*, 735, doi:10.3892/ol.2021.12996.
4. Alkasalias, T.; Alexeyenko, A.; Hennig, K.; Danielsson, F.; Lebbink, R.J.; Fielden, M.; Turunen, S.P.; Lehti, K.; Kashuba, V.; Madapura, H.S.; et al. RhoA knockout fibroblasts lose tumor-inhibitory capacity in vitro and promote tumor growth in vivo. *Proc Natl Acad Sci U S A* **2017**, *114*, E1413-E1421, doi:10.1073/pnas.1621161114.
